# Human Embryonic and Rat Adult Stem Cells with Primitive Endoderm-Like Phenotype Can Be Fated to Definitive Endoderm, and Finally Hepatocyte-Like Cells

**DOI:** 10.1371/journal.pone.0012101

**Published:** 2010-08-11

**Authors:** Philip Roelandt, Karen Ann Pauwelyn, Pau Sancho-Bru, Kartik Subramanian, Bipasha Bose, Laura Ordovas, Kim Vanuytsel, Martine Geraerts, Meri Firpo, Rita De Vos, Johan Fevery, Frederik Nevens, Wei-Shou Hu, Catherine M. Verfaillie

**Affiliations:** 1 Interdepartmental Stem Cell Institute Leuven, Catholic University Leuven, Belgium; 2 Hepatology Department, University Hospitals Leuven, Belgium; 3 Stem Cell Institute Minnesota, University of Minnesota, Minneapolis, Minnesota, United States of America; 4 Department of Chemical Engineering and Materials Science, University of Minnesota, Minneapolis, Minnesota, United States of America; 5 Pathology Department, University Hospitals Leuven, Leuven, Belgium; University of Southern California, United States of America

## Abstract

Stem cell-derived hepatocytes may be an alternative cell source to treat liver diseases or to be used for pharmacological purposes. We developed a protocol that mimics mammalian liver development, to differentiate cells with pluripotent characteristics to hepatocyte-like cells. The protocol supports the stepwise differentiation of human embryonic stem cells (ESC) to cells with characteristics of primitive streak (PS)/mesendoderm (ME)/definitive endoderm (DE), hepatoblasts, and finally cells with phenotypic and functional characteristics of hepatocytes. Remarkably, the same protocol can also differentiate rat multipotent adult progenitor cells (rMAPCs) to hepatocyte-like cells, even though rMAPC are isolated clonally from cultured rat bone marrow (BM) and have characteristics of primitive endoderm cells. A fraction of rMAPCs can be fated to cells expressing genes consistent with a PS/ME/DE phenotype, preceding the acquisition of phenotypic and functional characteristics of hepatocytes. Although the hepatocyte-like progeny derived from both cell types is mixed, between 10–20% of cells are developmentally consistent with late fetal hepatocytes that have attained synthetic, storage and detoxifying functions near those of adult hepatocytes. This differentiation protocol will be useful for generating hepatocyte-like cells from rodent and human stem cells, and to gain insight into the early stages of liver development.

## Introduction

Many groups are investigating alternative sources of functional hepatocyte-like cells to alleviate the shortage of human hepatocytes needed for cell replacement therapies and for pharmaceutical applications [1], [2], [3], [4], [5]. Candidates for the generation of hepatocytes range from endogenous liver progenitor cells, hematopoietic and stromal cells, to pluripotent cells, such as ESC (reviewed in [6]). Several differentiation protocols have been described to obtain hepatocyte-like cells from stem cells that used cytokines involved in mammalian liver development [7], [8]. However, few studies have addressed if differentiation to hepatocyte-like cells *in vitro* occurs via similar developmental steps as liver development *in vivo*, nor have they proven to be applicable to pluripotent cells from different species, or for adult stem cell differentiation.

Embryogenesis requires that the many differentiated cells generated from totipotent stem cells are correctly assembled in the different embryonic as well as extra-embryonic tissues. This occurs in a well orchestrated stepwise process, whereby totipotent stem cells are first fated to trophectoderm that differentiate solely to the extra-embryonic trophoblast, or to pluripotent cells in the inner cell mass (ICM)[9]. Cells within the ICM are subsequently fated to either primitive endoderm (PrE) which gives rise to parietal endoderm (PE) and visceral endoderm (VE) that, together with the trophoblast, contribute to extra-embryonic tissues, or epiblast cells, which give rise to the three germ layers of the embryo. During gastrulation, epiblast cells ingress in the primitive streak (PS) to form mesendoderm (ME) and definitive endoderm (DE)[10]. DE subsequently becomes fated to cells that ultimately populate the endodermal organs including the gut, pancreas, liver, and lungs. Theoretically, for a cell to become a terminally differentiated cell type, it must undergo all consecutive steps of development to be able to respond to the surrounding differentiation cues.

The hepatic differentiation protocol of human ESC we developed, employed cytokine cocktails to mimic the major steps of embryonic and fetal liver development, resulting in the sequential fating of ESC to PS/ME/DE, hepatoblasts and more mature hepatocytes. We also evaluated if this protocol would support hepatic differentiation of adult cell types with higher differentiation potential, such as rat multipotent adult progenitor cells (rMAPC). rMAPC are isolated clonally from cultured rat bone marrow (BM). rMAPC are characterized by the expression of genes associated with pluripotency such as *Oct4*, *Rex-1* and *Sall4* (but not *Nanog* and *Sox2*), genes used to generate iPSC such as *Klf2/4, n/c-Myc* and *Lin28,* but also genes typical for PrE, such as *Foxa2, Sox7, Sox17, Gata4, Gata6* [11], [12], [13]. Like mESC, rMAPC depend on leukemia inhibitory factor (LIF) to remain undifferentiated (manuscript in preparation). These partial similarities between rMAPC and ESC lead us to test if the protocol developed from hESC (and also suitable for mouse iPSC [14]), could be used to induce rMAPC differentiation to hepatocyte-like cells.

## Results

### Development of a four-step protocol for hepatocyte-like cell generation from human ESC

**Figure 1:**
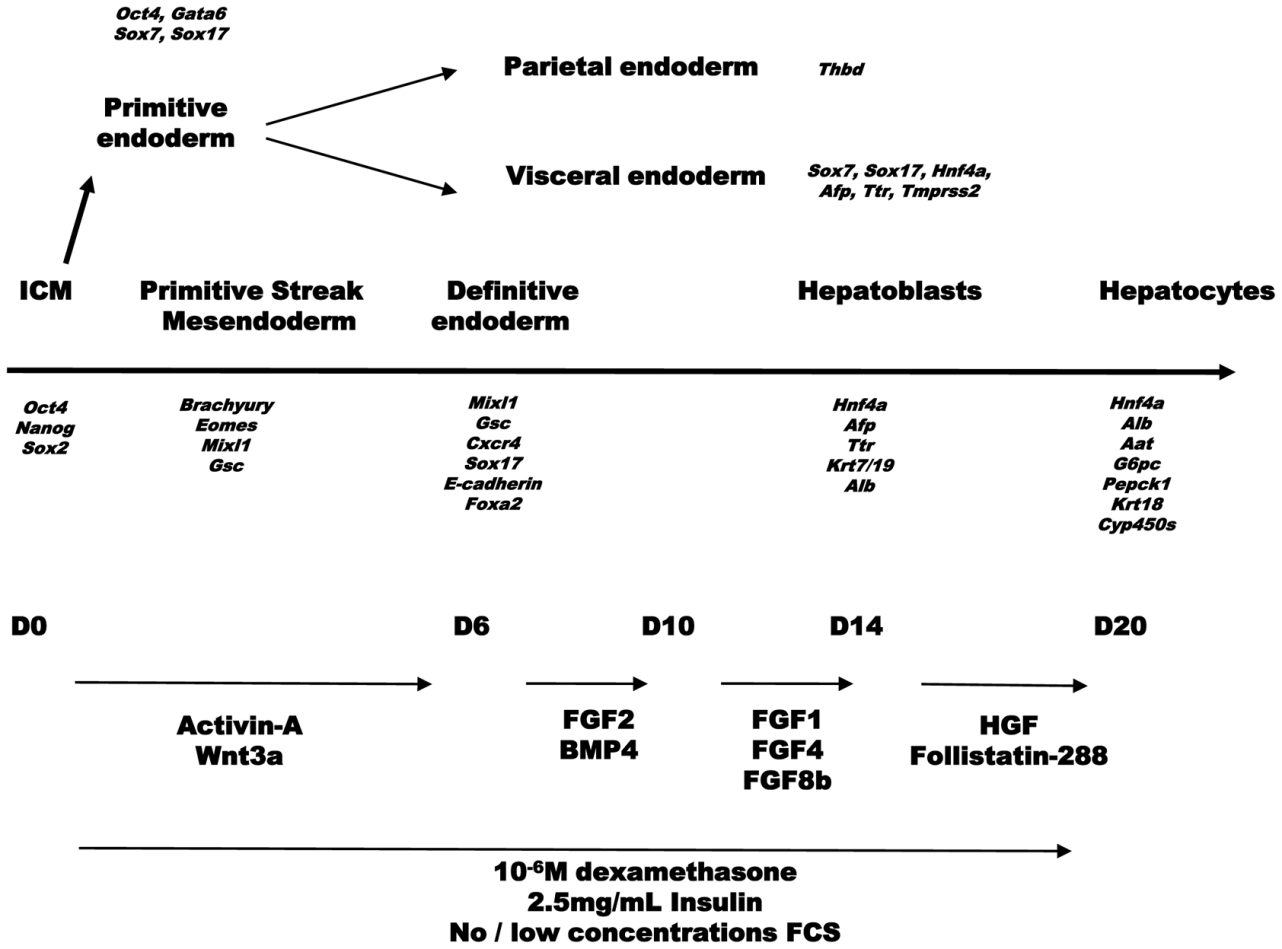
Overview of liver embryogenesis on which liver differentiation protocol is based. Genes specifically expressed at different steps during liver development are shown, in italics, under each step of lineage commitment. Cytokines used as well as other culture conditions are shown.

Many cytokines and signals that govern the sequential developmental steps have been identified from the study of model organisms. Based upon this information, we developed a 4-step differentiation protocol, mimicking four distinct steps of hepatic development (Figure 1). In step I we mimicked gastrulation by exposing cells to 100 ng/ml Activin-A, replacing the Nodal/Crypto signal [15], and 50 ng/ml Wnt3a [16], [17], [18]. In step II, 10 ng/ml FGF2 and 50 ng/ml BMP4 were added to specify DE to a hepatic fate, as these cytokines are secreted *in vivo* by the adjacent cardiac mesoderm and septum transversum mesenchyme, respectively [19], [20], [21], [22]. Step III was added as a proliferation and first maturation step of the newly specified early hepatoblasts and consisted of 25 ng/ml FGF8b, 50 ng/ml FGF1 and 10 ng/ml FGF4, based on the *in vitro* findings of Sekhon *et al* [23]. To induce a mature hepatocyte phenotype, step IV consisted of 20 ng/ml HGF and 100 ng/ml Follistatin-288, the first as a general hepatotrophic cytokine [24], the latter to favor hepatic over cholangiocyte differentiation [25]. In addition, 2.5 µg/ml insulin was added, as well as 1 µM dexamethasone, the latter to induce expression of mature hepatic specific genes [26].

**Figure 2:**
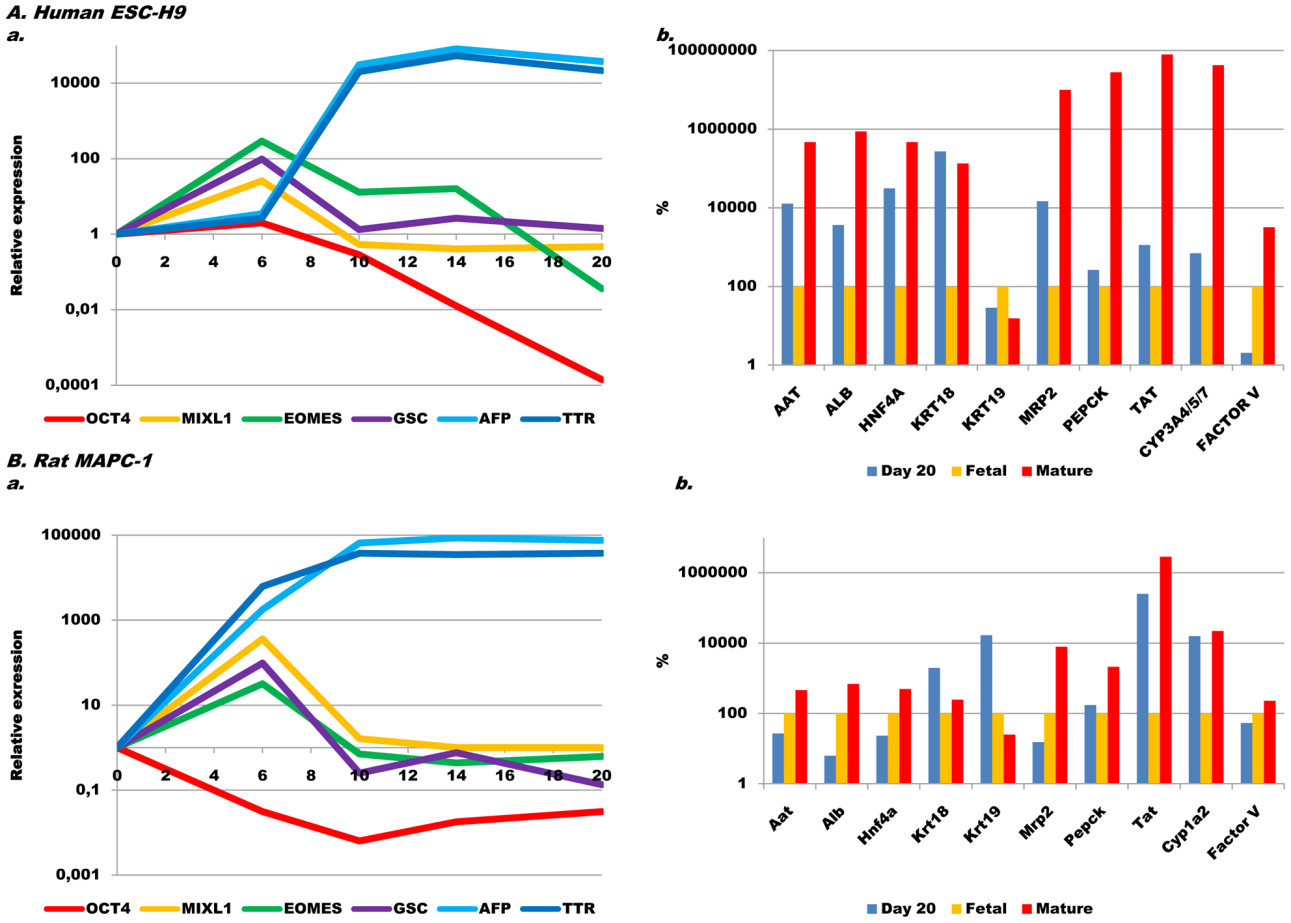
Sequential expression of genes found at different stages of endoderm and hepatic specification. Quantitative RT-qPCR was used to evaluate gene expression in (A) hESC-H9 and (B) rMAPC-1 during the 20 day differentiation process. Shown are relative expression values as compared to d0 on days 6, 10, 14 and 20 for genes in early liver development (n>3)(a) and relative expression to fetal hepatocytes (depicted as percentages and in comparison with mature hepatocytes) for more mature liver-specific genes (b).

Treatment of H9 (Figure 2A, Table S1A) and HSF6 (Table S1B) hESC with Activin-A/Wnt3a induced expression of the PS/ME/DE-specific genes (*GSC*, *BRACHYURY, EOMES, MIXL1, CXCR4)* [27], [28] by 2^2^–2^8^ fold, some peaking as early as d2 and others on d4 or d6 (Figure 2A/5A). Upon completion of step I (Activin-A/Wnt3a) on d6, expression of most of these PS/ME transcripts returned to baseline by d10. Transcripts of liver enriched transcription factors (LETF), known to govern liver development [22], [29], such as *PROX1, HNF1α, HNF1β, HNF4α, HNF6, FOXA2* were significantly induced during differentiation of both hESC lines. Consistent with this, the hepatoblast transcripts, *α-fetoprotein* (*AFP*) and *transthyretin* (*TTR*) became expressed between d6 and d10, reaching maximal expression levels at d14. *Albumin (ALB)* and *α_1_-antitrypsin (AAT)* transcripts increased gradually, becoming maximally expressed by d20, although levels were still below those of mature hepatocytes. Finally, a number of mature hepatocyte genes, including *cytochrome P450 isotypes (CYP3A4/5/7, CYP7A1), connexin-32 (CX32)*, *glucose-6-phosphatase (G6PC)* and *phosphoenolpyruvate carboxykinase (PEPCK1),* as well as coagulation associated genes (*FACTOR V, FACTOR VII*, *protein C* (*PROC*) and *γ-glutamyl carboxylase* (*GGCX*)) became maximally expressed between d14 and d20, but did not reach levels seen in mature hepatocytes (Figure 2A, Table S1A/B). With increasing differentiation, *OCT4* expression gradually decreased although low levels (0.017±0.004%) remained present at day 20. Although pancreatic cells are also derived from the ventral foregut endoderm, transcripts for *PTF1A*, *NKX6.1*, *PDX1* and *NGN3*, expressed in pancreatic progenitors and endocrine progenitors, were not up-regulated during hepatic differentiation (Table S1A/B).

**Figure 3:**
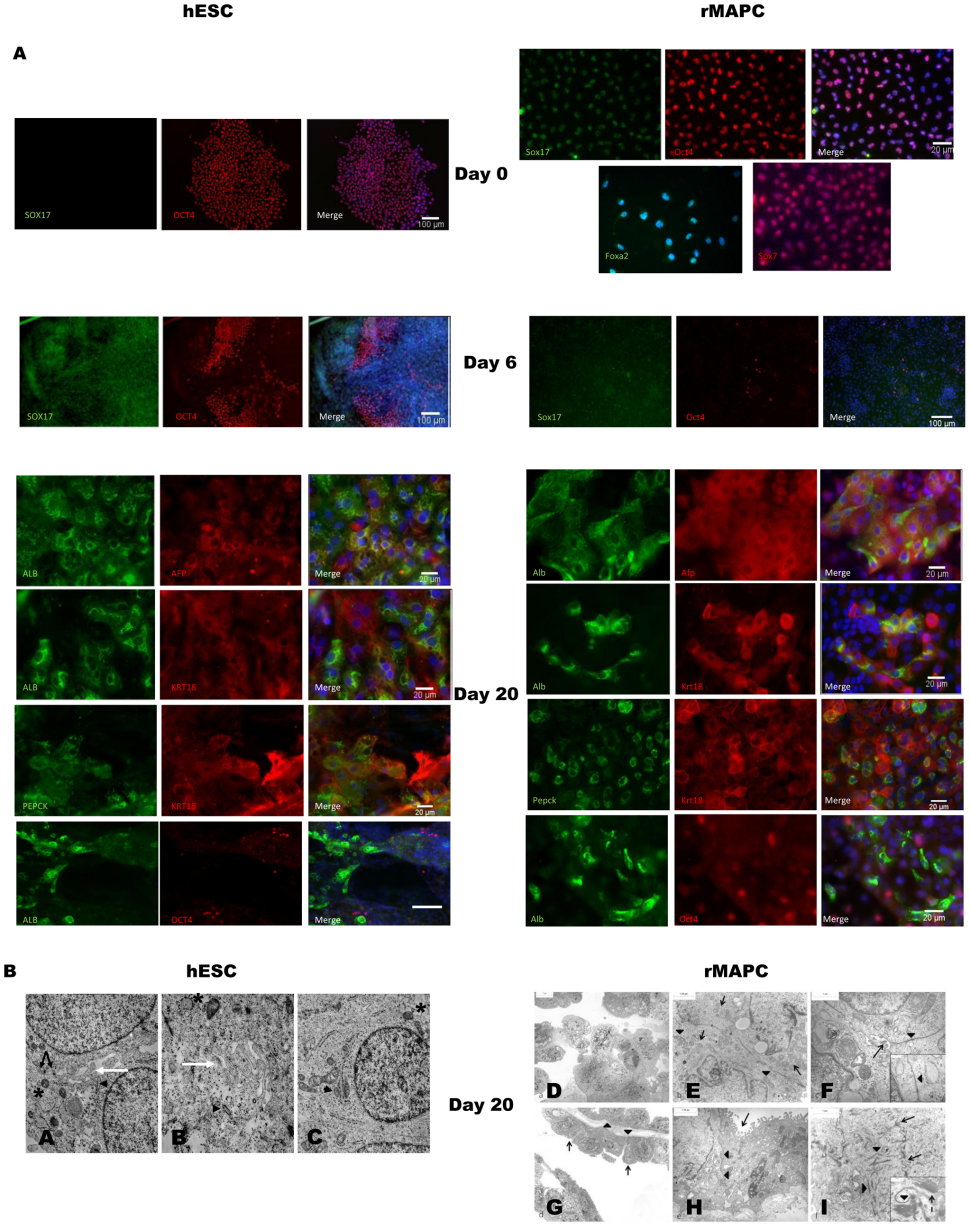
Immunofluorescence assessment of transcription factors, structural proteins and hepatoblast/hepatocyte specific proteins in hESC and rMAPC-1 and their progeny. [A] hESC-H9 and rMAPC-1 day 0, 6, 20. [B] Electron microscopy of d20- progeny of hESC-HSF6 and rMAPC-1. *Panels A–C: Differentiated hESC-HSF6 show characteristics of hepatocyte-like cells.* Shown are polarized hepatocyte-like cells with desmosomes (C, black arrow), bile canaliculi (A and B, white arrow), mitochondria (A–C, black asterisk) and rough endoplasmatic reticulum (A–C, black arrowhead). *Panels D–I: d20 rMAPC-1 progeny have characteristics of both hepatocyte-like cells (D–F) and bile duct-like cells (G–I).* Hepatocyte like cells (D–F): Some colony forming epithelial cells (D) contained many cytoplasmatic organelles as RER cisternae (E, arrow) and mitochondriae (E, arrow head), and formed with each other intercellular bile canalicular structures lined by microvilli (F, arrow) and sealed by junctional complexes (F, arrow head), even gap junctions were observed (F inset, arrow head). Bile duct-like cells (G–I): In addition, some epithelial cells arranged in layers and tubules presented at their apical pole many short microvilli (G and H, arrow) while their basal pole was surrounded by basement membranes (G, arrow head). The lateral membranes formed many interdigitations (H, arrow heads and I, arrow). In the cytoplasm a moderate amount of bundles of cytokeratin filaments (I, arrow heads) became obvious. Inset in I shows a bundle of cytokeratin filaments (arrow head) and a desmosome (dashed arrow).

We next investigated the percentage of hESC-H9 progeny that became committed to PS/ME/DE or hepatoblasts and hepatocytes using immunocytochemistry. The majority of undifferentiated hESC expressed OCT4 but not SOX17 (Figure 3A) or FOXA2 protein (data not shown). By d6, OCT4^−^/SOX17^+^ cells were found interspersed with remaining OCT4^+^ clusters (Figure 3A). On d20, dense colonies of ALB^+^ cells could be detected (Figure 3A). Although the density of ALB^+^ cells is highly variable within the well (range 5.6 to 49.3% in randomly taken pictures), on average 17.7±11.2% of the hESC-progeny were ALB^+^. Many of these cuboidal ALB^+^ cells still co-expressed AFP protein, consistent with a hepatoblast phenotype. A fraction of H9-progeny expressed ALB but no longer AFP; and areas of cytokeratin-18 (KRT18)^+^/PEPCK^+^ cells were found, both consistent with a mature hepatocyte phenotype (Figure 3A). Less than 1% of H9-progeny were still OCT4^+^ (Figure 3A). Ultrastructurally, hESC-HSF6 derived progeny contained medium sized and large epithelial cells with typical hepatocyte-like phenotype (Figure 3B).

**Figure 4:**
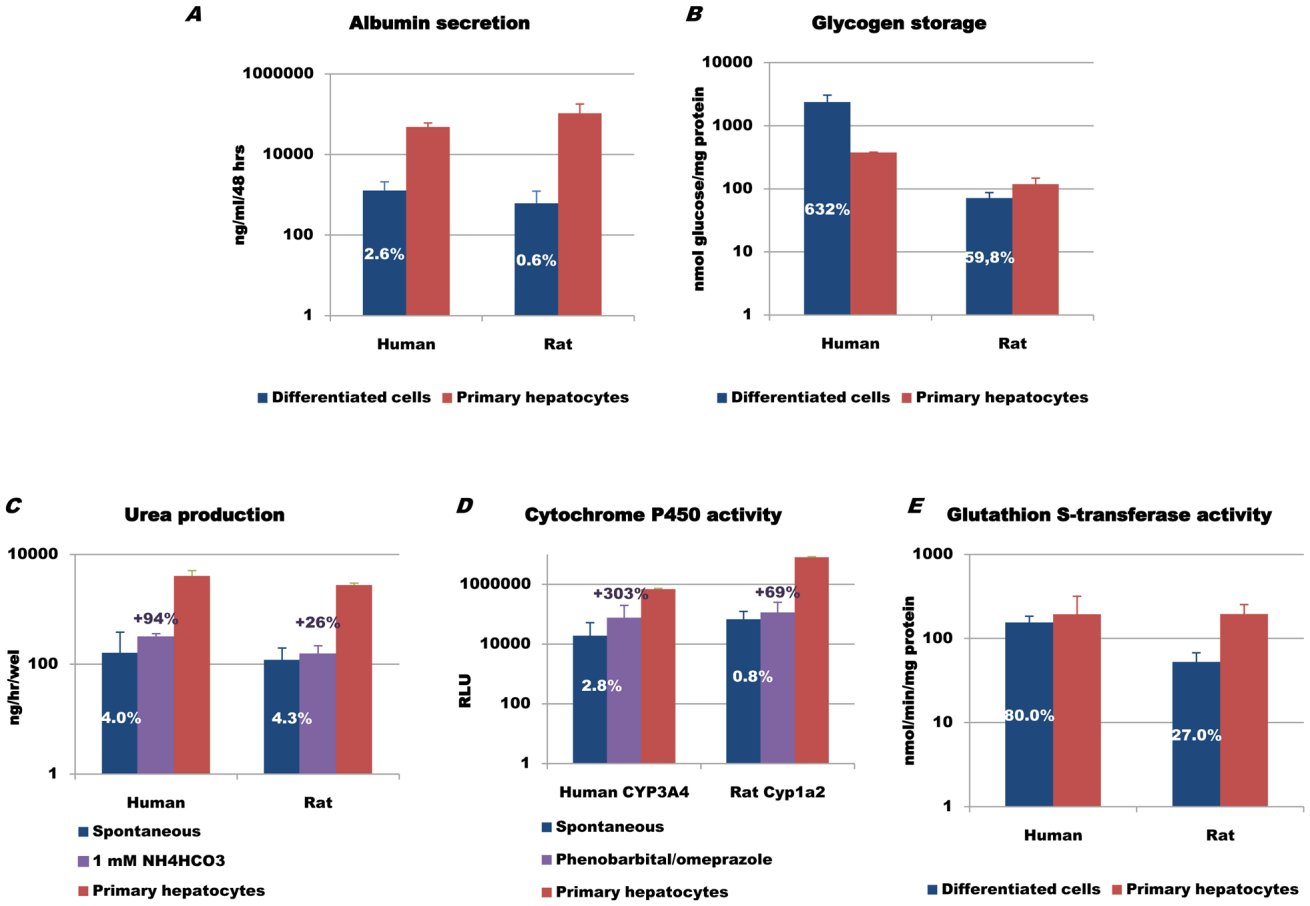
hESC-H9 and rMAPC-1 progeny display functional properties of hepatocytes on d20 (n >3). The following percentages are functional capacity of hESC- and rMAPC progeny compared to mature human and rat hepatocytes, respectively. [A] Albumin secretion (ng/mL/48 h), hESC 2.6%, rMAPC 0.6%. [B] Storage of glycogen (nmol glucose/mg protein), hESC 632%, rMAPC 59.8%. [C] Spontaneous (hESC 4.0%, rMAPC 4.3%) and NH_4_HCO_3_-stimulated urea production (hESC 7.7% (+94% induction), rMAPC 5.5% (+26% induction)) [D] Baseline (hESC 2.8%, rMAPC 0.8%) and induced cytochrome P450 activity (hESC 11.1% (+303% induction), rMAPC 1.4% (+69% induction)). 500 µM phenobarbital was used for induction of CYP3A4, while 10 µM omeprazole was used to induce Cyp1a2). [E] Glutathion S-transferase activity (nmol/min/mg protein), hESC 80.0%, rMAPC 27.0%.

We also assessed the ability of stem cell derived progeny to perform typical synthetic, storage and detoxification functions of hepatocytes. Human albumin was present in differentiating H9 and HSF6 supernatants from d14, reaching levels of 2.6 to 5.7% of mature human hepatocytes by d20 (Figure 4A, Table S2). Progressively more glycogen was stored in H9 progeny from d6 onwards to levels up to 6 times higher than (short term cultured) mature hepatocytes on d20 (Figure 4B). We also evaluated detoxification functions of the hepatocyte-like cells generated. In response to 1 mM ammonia, increased urea production was detectable from d14, becoming maximal on d20 (levels of 4.0% up to 7.7% after NH_4_HCO_3_ compared with mature hepatocytes)[30] (Figure 4C). Expression of *CYP7A1* (*cholesterol 7-α-hydroxylase*), which is the rate-limiting enzyme in the synthesis of bile acid from cholesterol, increased in H9 and HSF6-progeny by >2^7^ fold by d20, reaching levels 10-fold lower than in mature hepatocytes (Table S1A-B). RT-qPCR, using primers that recognize *CYP3A4/5/7* transcripts (phase 1 enzymes), demonstrated that *CYP3A4/5/7* expression levels in H9/HSF6 d20-progeny were higher expressed compared to fetal human hepatocytes (Table S1A–B). In addition, both H9 and HSF6 progeny expressed *CYP3A7* at levels found in postnatal liver. CYP3A4/5/7 activity was detected in d20-hESC-H9 progeny, which was inducible by 500 µM phenobarbital (levels of 2.8% up to 11.1% after phenobarbital compared with mature hepatocytes)(Figure 4D). Differentiated hESC also expressed phase 2 enzymes involved in sulphation and glucuronidation, such as UDP-glucuronidation (*UGT1A1*) and glutathione-S-transferase (*GST*) (Table S1A–B). Moreover, glutathion S-transferase activity of d20 H9 progeny reached levels of 80% of mature hepatocytes (Figure 4E). Hence, hESC progeny displayed many of the functional properties of mature hepatocytes, some at 5–10% of mature hepatocytes, others even higher than primary hepatocytes, despite the fact that only approximately 20% of the mixed cell population were ALB^+^.

Although some studies have concluded that differentiation of hESC leads to a more homogeneous population of albumin protein expressing hepatocyte-like cells than we demonstrate here, the functional properties we demonstrate for non-purified hESC progeny, including albumin secretion [31], [32], [33], [34], [35], urea secretion [34], [35], [36] and cytochrome P450 activity [33], [34], [36], [37] are in line, or even more robust than observed in reportedly more homogeneous progeny. However, as the latter were in general not compared with primary hepatocytes, such comparison is not fully possible. In addition, many published studies perform only minimal functional characterization of the hESC progeny in vitro, making comparisons not readily possible.

### Similar sequential activation of PS/ME/DE, hepatoblast and hepatocyte genes in rat multipotent adult progenitor cells

As rMAPC have a number of features in common with pluripotent stem cells, such as high levels of *Oct4, Rex1, Lin28, Sall4, Klf2,4,5* expression, dependence of LIF and apparent differentiation to cells of the three germ layers [11], [12], we hypothesized that the protocol developed for hESC, which also induces hepatic differentiation of mouse induced pluripotent stem cells [14] may also induce hepatocyte-like cell differentiation from rMAPC [38]. Two independently isolated clonal rMAPC lines (rMAPC-1 and rMAPC-2) were used (Figure 2B, Table S1C–D).

A 2^5^ to >2^10^ fold increase in transcripts for PS, ME and DE specific genes (*Gsc*, *Eomes, Mixl1, Cxcr4)* was induced on d6 by culturing rMAPC-1 and rMAPC-2 at high cell density and with Activin-A/Wnt3a. Like in hESC, levels of these transcripts returned to near baseline following completion of step 1. We also found a significant (p<0.05) induction of the LETFs, *Hnf4α, Hnf1α* and *Prox1*, while expression of *Hnf1β* and *Foxa2*, already high in undifferentiated rMAPC, remained nearly unchanged over the 20-day differentiation period. Expression of *Afp* and *Ttr* transcripts was highly induced from d6 on, reaching maximal levels on d10 and persisted until d20. Expression of *Alb* and *Aat* increased gradually by >2^15^ fold by d20 and d14, respectively. Expression of *Arg1, bile salt export pump (Bsep)*, *multidrug resistance-associated protein 2 (Mrp2), G6pc, Tat and Pepck1* increased slowly throughout differentiation to levels still lower than in mature rat liver (Figure 2B, Table S1C–D). Transcripts for the coagulation associated genes, *Factor V, Ggcx* and *Proc* increased 2^4^–2^10^ fold, while *Factor VII* transcripts were not induced. Hepatic maturation was accompanied by decreased, but persistent expression of *Oct4* (2.2±1.2%). As was seen for hESC, we found only a minimal induction of *Pdx1*, and transcripts specific for pulmonary epithelium, such as *Sftp-a* and *Nkx2.1* were not induced (data not shown). Identical results were found for 2 additional rMAPC lines (Figure S1). However, when rat BM cells isolated under MAPC conditions that do not express *Oct4* and the PrE transcripts (rClone-1) [11], [12], were subjected to the same differentiation protocol, only very limited hepatic gene expression was found (data not shown).

**Figure 5:**
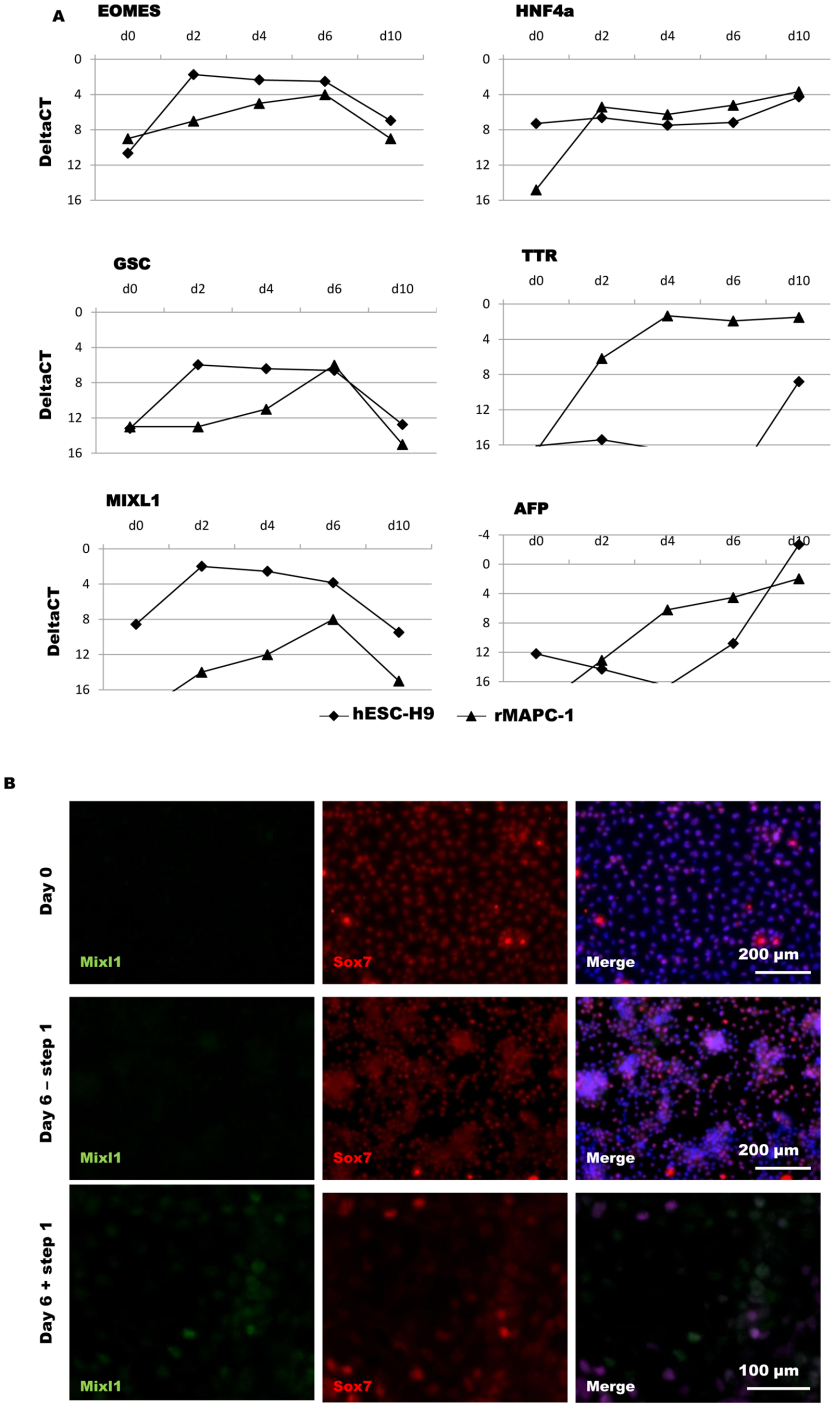
Time course analysis of PS/ME/DE and visceral endoderm gene expression during differentiation of rMAPC and hESC. [A] Quantitative RT-PCR was used to evaluate the time point of maximal expression of transcripts of genes expressed in PS/ME/DE (*Eomes*, *Gsc*, *Mixl1*) and visceral endoderm genes (*Hnf4a*, *Ttr, Afp)* in rMAPC and hESC. Shown are mean DeltaCT values (n≥3). [B] Immunofluorescence assessment of rMAPC-1 progeny by staining for Mixl1 (green)/Sox7 (red) on d0, d6 without cytokines (- step1) and d6 with cytokines (+ step1) (Activin-A/Wnt3a).

Consistent with the rMAPC transcriptome data [11], nearly 100% of undifferentiated rMAPC-1 expressed Oct4, Sox17, Foxa2 and Sox7 (Figure 3A) but not Mixl1 (Figure 5B), Afp, Alb or Pepck (data not shown). On d6, most rMAPC-1 progeny were Oct4^−^, but remained Sox17^+^, compatible with differentiation to ME/DE. By d20, approximately 80% of rMAPC-1 progeny expressed Afp, while expression of Alb was variable and patchy, similar to what we found for hESC progeny (range 1.3 to 53.4%, average 11.7±16.6% Alb^+^) (Figure 3A). Of the Alb^+^ cells, a small proportion did no longer express Afp, and we detected areas of Pepck^+^/Krt18^+^-cells, both consistent with a mature hepatocyte phenotype (Figure 3A). On d20, 1–5% of rMAPC-1-progeny were still Oct4^+^ (Figure 3A). Ultrastructurally, rMAPC-1 derived progeny presented as clusters of polarized medium sized and large epithelial cells. The more compact clusters and colony forming epithelial cells showed a typical hepatocyte-like phenotype. The epithelial cells arranged in layers and tubules presented characteristics of bile duct cells (Figure 3B).

rMAPC progeny also had functional characteristics of mature hepatocytes. Albumin was secreted in culture supernatants of rMAPC-1 and rMAPC-2 from d14 onwards, with levels of 0.6 to 3.3% of mature rat hepatocytes by d20 (Figure 4A, Table S2). rMAPC-1 and rMAPC-2 stored glycogen from d6 onwards reaching levels of 59.8% of mature hepatocytes by d20 (Figure 4C). Like in hESC-progeny, spontaneous urea production was found in rMAPC-1 progeny, while urea production in response to ammonia (urea cycle) was detectable from d10, becoming maximal on d20 (levels of 4.3% up to 5.4% after NH_4_HCO_3_ compared to mature hepatocytes) (Figure 4C). Finally, we demonstrate that d20 rMAPC-1 progeny expressed functionally active Cyp1a2 (phase 1) inducible with 10 µM omeprazole (levels of 0.8% up to 1.4% after omeprazole compared with mature hepatocytes) (Figure 4D) and 27% glutathion S-transferase activity (phase 2) (Figure 4E). Thus, as observed for hESC, rMAPC progeny displayed many of the functional properties of mature hepatocytes at 1–60% of mature hepatocytes, even if only approximately 12% of the mixed cell population were Alb^+^.

### Differences in PS/DE and PE/VE gene expression consistent with putative developmental stage of hESC and rMAPC

All rMAPC lines used express high levels of *Oct4* but not *Nanog* and *Sox2.* As they also express *Sox7, Sox17, Gata4, Gata6* and *Foxa2*, and minimal or no *Mixl1*, *Hnf4α, Afp* and *Ttr* transcripts, this phenotype is similar to that of ICM cells that have suppressed expression of *Nanog* and become PrE [39], [40], as well as of rat derived PrE cell lines (XEN-P cells) [41]. When expanded in the absence of LIF, rMAPC-1 very quickly lose expression of *Oct4*, with a concomitant fast increase in the VE transcripts, *Hnf4α, Afp* and *Ttr* (manuscript in preparation). To verify whether the differentiation protocol induced rMAPC commitment to intra-embryonic tissue, DE, and then hepatic cells, or merely to VE or PE, which also express a number of presumed hepatic genes [39], we further analyzed the initial steps of differentiation of rMAPC and compared this with the initial steps of hESC differentiation.

When rMAPC were cultured at high density with Activin-A/Wnt3a but without LIF, a very fast induction of *Hnf4α* expression occurred, a transcription factor found in VE but not PE, with maximal expression (2^7^ fold induction) as early as after 12 hours (data not shown). In addition, *Ttr* transcripts increased 2^10^ and 2^15^ fold on d2 and d4, respectively, and *Afp* transcripts 2^6^ and 2^13^ fold, respectively, also consistent with differentiation to VE. By contrast, for hESC, maximal expression of *HNF4α, TTR* and *AFP* was not detected until d10 (Figure 5A, Table S1A–B). *Sox7*, typically expressed in PrE and VE, was expressed in undifferentiated rMAPC and remained expressed throughout differentiation. Although some increase in *SOX7* transcript levels was found on d2 for hESC, the most pronounced increase was seen around d10 of differentiation (data not shown). As *SOX7* is also expressed later during development in tissues such as lung, liver, and a number of mesodermal cell types [42], the increase in *SOX7* levels in hESC may not represent differentiation towards VE/PE. Likewise, the VE transcript *Tmprss2*, was induced by >2^10^ fold on d6 in rMAPC-1, and persisted throughout differentiation while no increase in transcript levels for *thrombomodulin* (*Thbd*), characteristic for PE [39], was found (Table S1C). Hence, the conditions used for hepatic differentiation of rMAPC appeared to induce differentiation from a PrE to a PE phenotype.

We demonstrate, however, also conclusively that upon exposure to Activin-A/Wnt3a, d6-rMAPC expressed 2^4^ to 2^10^ fold higher levels of the PS/ME/DE gene transcripts *Eomes, Mixl1, Lhx1*, *Tm4sf2*, *Cxcr4* and *Gsc*, and expressed *Brachyury* (Figure 2B, Table S1C–D). Noteworthy, maximal expression of these PS/ME/DE genes in rMAPC-progeny was found on d6, 4 days later than in hESC (Figure 5A). The fast induction of *MIXL1*, *EOMES* and *GSC* by d2 in hESC is consistent with the notion that hESC are similar to epiblast cells and rapidly express PS genes upon differentiation (Figure 5A). The PS/ME/DE commitment of rMAPC is dependent on Activin-A/Wnt3a, as culture of rMAPC without LIF but also without Activin-A/Wnt3a, or with 1 ng/mL rather than 100 ng/mL Activin-A induced *Mixl1, Cxcr4, Gsc* and *Tm4sf2* significantly less (p<0.05) (Table S3).

To further demonstrate that at least some of the rMAPC can be fated to intra-embryonic endodermal cells, we stained rMAPC-1 progeny on d6 for Sox7 and Mixl1 (Figure 5B), as a Sox7^−^/Mixl1^+^ phenotype is compatible with a PS/ME/DE phenotype. Consistent with RT-qPCR results, no Mixl1^+^ cells were found in the absence of Activin-A/Wnt3a, while most cells remained Sox7^+^. In cultures containing Activin-A/Wnt3a, approximately 5% of d6 rMAPC-1 expressed Mixl1 but not Sox7. As nearly 100% of undifferentiated rMAPC-1 co-expressed Oct4, Sox17, Foxa2 (Figure 3A) and Sox7 (Figure 5B) proteins, this suggests that cells with a PrE-like phenotype can still be induced to differentiate to PS/ME/DE.

## Discussion

Using insights from mammalian liver development, we developed a differentiation protocol to generate hepatocyte-like cells from hESC by sequential induction of primitive streak/mesendoderm/definitive endoderm, followed by gradual hepatic maturation. The same protocol, with omission of FCS, can also be used to induce differentiation of rMAPC.

Two distinct populations of endoderm are sequentially induced during mouse development. Concomitant with implantation (E4.5), the PrE delaminates from the surface of the blastocoele of the inner cell mass (ICM). In contrast to PrE, cells fated to become DE, are derived from epiblast cells which ingress the PS during the process of gastrulation. DE is then specified to hepatic endoderm, hepatoblasts and finally mature hepatocytes in response to a series of factors, some secreted by surrounding cells [43]. As both types of endoderm express many transcripts in common (*Foxa2*, *Gata4*, *Gata6*, *Sox17, Hnf4α, Afp*, *Ttr*, …), one should take into account the minimal differences in transcript and protein expression between these two types of endoderm when investigating *in vitro* differentiation of pluripotent stem cells towards hepatic endoderm. Thus, monitoring the sequential and transient expression of a complement of genes/proteins consistent with PS/ME, during the course of the *in vitro* differentiation process can help to distinguish between DE and PrE. Although some of the PS/ME/DE genes assessed here, are also expressed in VE (i.e. *Mixl1*, *Gsc*) or trophectoderm (i.e. *Eomes*) or later during development (i.e. *Cxcr4*), the transient up-regulation of all of these genes together in response to Activin-A and Wnt3a and their subsequent down-regulation upon withdrawal of these cytokines in both hESC and rMAPC, strongly indicates transition through a PS-like intermediate prior to acquisition of hepatic characteristics. These findings at the transcriptome level were also substantiated at the protein level. It should be noted that even though we demonstrate that at least 5% of rMAPC differentiate to Sox7^−^/Mixl1^+^ DE cells, another fraction of rMAPC may differentiate towards VE, given the very early rise of VE genes such as *Tmprss2*, *Afp, Ttr* and *Hnf4α*, and persistent expression of Sox7 protein on d6. In contrast, no substantial up-regulation of *SOX7, AFP, TTR* and *HNF4α* was found before d10 in hESC. In addition, the highest levels of PS/ME/DE gene expression was obtained at an earlier time point in hESC compared to rMAPC, consistent with the notion that hESC are developmentally comparable with epiblast/embryonic ectoderm cells, the stage just prior to gastrulation, while we hypothesise that rMAPC first have to switch fate from a PrE-like phenotype to epiblast-like cell, thereby gaining the ability to undergo “gastrulation”.

It is at first sight surprising that cells with a phenotype consistent with PrE can be fated to intra-embryonic cells (PS/ME/DE) and hepatocyte-like cells. It is commonly believed that DE cells derived from the epiblast are the sole cells that, following gastrulation, give rise to epithelium of the digestive tract, pancreas, liver and lungs, whereas VE is derived from the subpopulation of *Nanog* ^-^ cells in the ICM. However, a recent lineage tracing study in early embryos *in vivo* has demonstrated that this dogma may not be true [44], [45]. Kwon *et al* demonstrated that VE cells can, at the time of gastrulation, intersperse with epiblast derived cells, enter the embryo proper, proliferate and contribute to 10–40% of the epithelium of the foregut, midgut and hindgut of 12–18 somite embryos [44]. Even though the study did not address whether these VE cells themselves are patterned to cells of PS, ME and then DE, they found down-regulation of the VE marker, *HNF4α*, in the distally positioned VE-derived cells that are eventually incorporated in the embryo proper. There is also mounting evidence that fate decisions early during development between PrE and epiblast are metastable. For instance, expression levels of *Nanog* in ESC and the ICM can fluctuate, where *Nanog^low^* cells may start to re-express *Nanog*, allowing differentiation not to VE and PE, but also intra-embryonic cell types [46], [47]. In contrast to the findings that cells initially expressing *Oct4, Sox17, Foxa2* and *Sox7* can be fated to PS/ME/DE and further to hepatocyte-like phenotype, Séguin et al, recently demonstrated that forced expression of *SOX7* in hESC results in the commitment of hESC to cells with a phenotype similar to extra-embryonic endoderm (XEN) cells, which still co-express *NANOG* and *OCT4*. In contrast to rMAPC, SOX7-hESC could not be induced to differentiate to cells with hepatic or pancreatic characteristics [48]. As *SOX7* decreased Wnt/β-catenin-stimulated transcription [42], it is possible that the inability to suppress the constitutively expressed *SOX7*, which may prevent activation of β-catenin via Wnt3a signaling, is responsible for the absence of PS/ME/DE induction. How rMAPC exactly can commit to cells expressing PS/ME/DE transcripts, why some rMAPC differentiate to VE and others apparently to PS/DE and finally, whether PrE cells derived from rat blastocysts, like XEN-P cells [41] can be fated to PS/ME/DE and hepatic endoderm using this protocol, will require further evaluation.

A prerequisite for the further activation of the hepatic gene program is the significant induction of LETFs. As a combination of these LETFs became expressed at substantial levels, hESC and rMAPC progeny should be poised to differentiate further towards mature hepatocytes, if supplied with additional proper cues. Indeed, following exposure to 3 additional steps, cells with different levels of maturation emerged by the end of the differentiation protocol, as evidenced by the significant expression of post-natal hepatic specific markers (*G6p, Cx32, Cyp’s, Pepck*), combined with a maintained expression of genes expressed in more primitive hepatic cells, such as *Afp*, which normally rapidly decreases at birth and is no longer expressed in mature hepatocytes. Indeed, most hESC and rMAPC-progeny stained for Afp at the protein level, some in co-expression with Alb, while only a minor fraction was Alb^+^/Afp^−^.

Although the hepatocyte-like cells derived from both hESC and rMAPC display several hepatic functions, such as albumin secretion, urea production, glycogen storage, Cyp450 and GST activity, levels were in general still approximately 5 to 10-fold lower than in mature hepatocytes. Thus, despite the use of cytokine cocktails known to play a role during liver development, the *in vitro* culture system still does not recreate all signals present *in vivo* that govern a coordinated maturation from pluripotent cells to terminally differentiated mature hepatocytes. As organogenesis does not solely depend on soluble factors but also cell-cell interactions, development of 3-dimensional culture systems wherein the anatomical features of developing liver lobules are recreated and the use of bio-reactors making it possible to more closely control physiological parameters such as pH and glycemia, may be needed to allow the creation of hepatocytes with fully mature characteristics and functions.

## Materials and Methods

### Experimental procedures

All isolations were approved by the ethical committee for the use of human subjects in research and the ethical committee for use of animals in research of University of Minnesota, USA and Catholic University of Leuven, Belgium.

### Media Composition and Cytokines

#### Basal differentiation medium

60% DMEM-low glucose (Gibco 31885), 40% MCDB-201-water (Sigma M-6770), 0.25X Linoleic acid – Bovine serum albumin (LA-BSA) (Sigma L-9530), 0.25X Insulin-transferrin-selenium (ITS) (Sigma I-3146), 100 IU/mL Penicillin, 100 µg/mL Streptomycin (Cellgro 30-002-CI), 0.1 µM L-Ascorbic Acid (Sigma A8960), 10^−3^ µM Dexamethasone (Sigma D2915), 55 µM 2-mercaptoethanol (Gibco 31350).

#### Human ESC expansion medium

80% DMEM/F-12 (Gibco 21331), 20% Knockout Serum Replacement (Gibco 10828), 2 mM L-glutamine (Invitrogen 25030), 0.1 µM MEM non-essential amino acids (NEAA; Invitrogen 11140), 0.1 µM 2-mercaptoethanol, 4 ng/mL FGF2.

#### Mouse embryonic fibroblasts (MEFs) expansion medium

90% DMEM high glucose (Gibco 21063), 10% FCS (Hyclone), 2X L-glutamine, 2X penicillin-streptomycin, 2X MEM NEAA, 110 µM 2-mercaptoethanol.

#### Rat MAPC expansion medium

60% DMEM-low glucose (Gibco 31885), 40% MCDB-201-water, 2% FCS (HyClone CH30160.03), 1X Linoleic acid – Bovine serum albumin (LA-BSA), 1X Insulin-transferrin-selenium (ITS), 100 IU/mL Penicillin, 100 µg/mL Streptomycin, 0.1 µM L-Ascorbic Acid, 5×10^−8^ µM Dexamethasone, 55 µM 2-mercaptoethanol.

#### Cytokines

The following cytokines and growth factors (all from R&D Systems) were added for cell expansion or during differentiation: rh/m/rActivin-A (338-AC), rhBMP4 (314-BP), rhFGF1(232-FA), rhFGF2 (233-FB), rmFGF8b (423-F8-025), rhFGF4 (235-F4-025), rmFollistatin-288 (769-FS), rmWnt3a (1324-WN), rmOncostatin M (495-MO), rhOncostatin M (295-OM), rhHGF (294-HGN), hPDGF-BB (220-BB). mEGF was from Sigma (E-1257) but, due to supplier problems, rmEGF (R&D Systems 2028-3G) was used subsequently.

### Cell Line Isolation and Maintenance

#### Mouse embryonic fibroblasts

MEFs were derived at the U. of Minnesota from E13-E14 CF-1 mice (Charles River Laboratories, Wilmington, MA) or purchased from Global Stem Inc, Rockville, USA. MEFs were maintained in MEF expansion medium and immortalized with Mitomycin C (KYOWA Mitomycin 2 mg). Mitomycin-treated MEFs were plated at a density of 35000 cells/cm^2^ on 0,1% gelatine (Ultrapure water 0,1% gelatine, Chemicon ES-006-B) coated 6 well plates.

#### Human embryonic stem cells

The HSF6 cells (obtained from Dr. M. Firpo, U. of Minnesota) and H9 cells (purchased from WiCell, Madison, WI) were cultured as described [49] on mitomycin-inactivated MEFs in hESC expansion medium. hESC were maintained in a 21% O_2_ – 5% (H9) or 10% (HSF6) CO_2_ – 37°C incubator and passaged 1:3 using collagenase IV (Gibco 17104) every 3–7 days.

#### Rat multipotent adult progenitor cells (MAPCs)

Isolation, characterization and maintenance of rMAPC-1, was described previously [11], [12], [13]. The rMAPC-2, rMAPC-3 and rMAPC-4 lines were isolated from bone marrow of E18 to 3 week old Fisher rats, using methods identical to those used for rMAPC-1.

### Hepatocyte Differentiation Culture

All differentiations were done in 12 or 24 well plates (Corning 12 wells 3513, 24 wells 3526) pre-coated with 2% Matrigel (BD 356231) diluted in PBS (Gibco 10010) for 1–2 h at 37°C, in a 21% O_2_ – 5.8% CO_2_ – 37°C incubator.

hESC were allowed to grow in feeder-conditioned hESC medium for 24 hours or until 50–70% confluent. To induce differentiation, expansion medium was switched to basal differentiation medium, supplemented with the sequential cytokine cocktails as described in Figure 1 for 20 days, and 2% FCS between d1-6 and 0.5% FCS between d6-20.

rMAPC were differentiated using the same protocol, except that the differentiation was done without FCS and the starting cell density was 50,000 cells/cm^2^ (as described in [38]).

### Evaluation of Liver Differentiation Protocol

As positive controls for functional tests rat primary hepatocytes were isolated from 5 weeks old Fischer rat using a two-step collagenase-perfusion method [50]. Human primary hepatocytes were purchased from Biopredic International (HEP220).

#### RT-qPCR

For RNA isolation, the RNeasy Mini-kit/Micro-kit (Qiagen 74104 and 74004) was used. DNAse treatment was performed using Turbo DNAse kit (Ambion 1907). cDNA synthesis was performed from 1 µg of RNA with Superscript III First-Strand synthesis system (Invitrogen 18080-051). Real time PCR was performed with SYBR Green Platinum SYBR green qPCR Supermix-UDG (Invitrogen 11733-046) in an Eppendorf realplex/ABI 7000 (Eppendorf) equipment. Relative gene expression was calculated by the 2^(−ΔΔCt)^ method compared to undifferentiated cells (day 0), using *GAPDH*/*Gadph* as housekeeping gene. Supplementary tables are represented as DeltaCt (ΔCt) values compared to *GAPDH*/*Gapdh* expression. ΔCt values >16 were considered not expressed (NE). The list of primers used can be found in Table S4. Fetal (E15) and mature rat liver was used to extract RNA as positive controls. RNA from fetal (third trimester) and mature human hepatocytes was a kind gift of Prof. M. Ott (Hannover Medical School, Germany).

#### Immunofluorescence

Differentiations were done in 4 well chamber slides (Nunc 177437) or in 12 well plates (Corning). Cells were fixed using 10% Neutral Buffered Formalin (NBF) for 15 minutes at room temperature (RT). Permeabilization was done for 15 minutes using PBS containing 0.2% Triton X-100 (PBST) (Acros Organics 422355000). PBST, containing 3% Normal Donkey Serum (Jackson, JACK017-000-121), was used for blocking for 30 minutes at RT. The cells were then incubated with the mixture of primary antibodies diluted in PBS containing 3% donkey serum and incubated overnight at 4°C. After three washes in PBS, the cells were incubated with the mixture of respective Alexa dyes conjugated secondary antibodies and Hoechst dye (Sigma 33258) for 30 minutes at RT. After final washes the cells were mounted with a cover slip at the top of Prolong® Gold mountant (Invitrogen P36930). All dilutions were optimized on both positive control cells (HepG2 for human cells, primary rat hepatocytes for rat cells) and negative control cells (Mouse Neural Stem Cells for early markers, undifferentiated rMAPC for liver-specific markers) and using the respective isotype control antibodies. The list of primary and secondary antibodies used can be found in Table S5. Quantification of albumin-positive cells was performed using Zeiss AxioVision Software version 4.8.1 on >10 randomly taken pictures.

#### Transmission electron microscopy (TEM)

TEM was performed on d20 rMAPC-1 and HSF6 progeny. Cells were washed twice with PBS and scraped to obtain cell clusters. The fragments were immediately fixed in 2.5% glutaraldehyde and 0.1 mol/L of phosphate buffer and stored at 4°C. After post-fixation in 1% osmium tetroxide and 0.1 mol/L of phosphate buffer, the samples were dehydrated in graded series of alcohol and embedded in epoxy resin. Ultrathin sections were cut, stained with uranyl acetate and lead citrate, and examined using a Zeiss EM 900 electron microscope (Oberkochen, Germany).

#### Albumin secretion

Rat and human albumin was measured using a quantitative ELISA kit (Starters Kit Bethyl E101 and respectively Bethyl E110-125 & E80-129) as per manufacturer's protocol.

#### Glycogen storage

Glycogen content was measured according to the spectrophotometrical method of Seifter *et al* [51].

#### Urea production

rMAPC and hESC progeny were washed with PBS and cultured with 1 ml of differentiation medium containing 0 or 1 mM NH_4_HCO_3_ for 24 hours. Urea content was calculated using QuantiChrom™ Urea Assay Kit (BioAssay Systems DIUR-500).

#### Cytochrome P450 activity

Cytochrome P450 subtype activity was detected by using the non-lytic method of P450-Glo™ Assay (Promega V8901 and V8771). Induction of CYP3A4 was performed by incubation with 500 µM phenobarbital, induction of Cyp1a2 by incubation with 10 µM omeprazole, as per manufacturer's protocol.

#### Glutathione S-transferase (GST) activity

Total GST activity was measured according to the spectrophotometrical method of Habig *et al* [52] using 1-chloro-2,4-dinitrobenzene (CDNB). The reaction mix contained 1 mM GSH and 1 mM CDNB in 0.1 M potassium phosphate buffer, pH 6.5. The reaction was started by adding 125 µl of sample, and the rate of formation of CDNB-GSH conjugate was monitored at 340 nm for 6 min. GST activity was calculated using the extinction coefficient of 9.6 mM^−1^ cm^−1^, and expressed as µmol of CDNB-GSH conjugate formed per min per mg of cellular protein. Total protein content was measured using the method by Bradford [53] with bovine serum albumin as standard protein.

### Statistics

Student's T-test was used for statistical analysis. A minimum of three independent experiments was performed for every described test.

## Supporting Information

Figure S1 Quantitative RT-PCR method was used to evaluate expression levels of Oct4, the primitive/visceral endoderm specific gene, Sox7, and levels of some genes expressed during PS/ME/DE (Mixl1, Cxcr4), and hepatoblast/hepatocyte commitment (Afp, Alb, Tat and G6pc). Shown are mean DeltaCT values of >3 for rMAPC-1 (data also in Figure 2 and Table S1) and rMAPC-2 (data also in Table S1), and two additional cell lines (n = 1) different experiments on days 0, 6, 10, 14 and 20.
(0.30 MB TIF)

Table S1 RT-qPCR analysis of gene expression in undifferentiated human ESC H9 and HSF6 (S1A and S1B) and rat MAPC-1 and rMAPC-2 (S1C and S1D), and during differentiation towards hepatocyte-like cells, using the protocol described in Figure 1, as well as values in fetal (third trimester) and adult human hepatocytes, and fetal (E15) and adult rat liver. Shown are mean DeltaCT values + s.d. (n>3). NE  =  not expressed (DeltaCT >16). −  =  not assessed. # =  peak expression of MIXL1 on day 2, by day 6 expression already back to baseline. Some of these data are also shown in Figures 2 and S1.
(0.23 MB DOC)

Table S2 Albumin production by hESC-H9, hESC-HSF6, rMAPC-1 and rMAPC-2 was measured in culture supernatants of undifferentiated stem cells as well as at different time points of differentiation towards hepatocyte-like cells and in supernatants of primary hepatocytes. Data are shown as mean concentrations after 48 h (ng/ml/48 h) + s.d. (n>3). − =  not assessed. The mean values op hESC-H9 and rMAPC-1 are also shown in Figure 4A.
(0.05 MB DOC)

Table S3 RT-qPCR analysis of expression of PS/ME/DE genes on d0 and d6 in rMAPC-1 treated with no, 1 ng/ml or 100 ng/ml Activin-A. Data are all from paired experiments, and are shown as DeltaCT compared with Gapdh. Shown are mean DeltaCT values + s.d. (n >3, except * n = 2). # = p<0.05 versus 100 ng/ml Activin-A.
(0.06 MB DOC)

Table S4 Primer list.
(0.12 MB DOC)

Table S5 Immunohistochemistry antibodies.
(0.06 MB DOC)
