# Supplementary figures and images for: Human Embryonic and Rat Adult Stem Cells with Primitive Endoderm-Like Phenotype Can Be Fated to Definitive Endoderm, and Finally Hepatocyte-Like Cells
Source: PLoS One. 2010 Aug 11;5(8):e12101. doi: 10.1371/journal.pone.0012101 (PMC2920330; doi:10.1371/journal.pone.0012101)

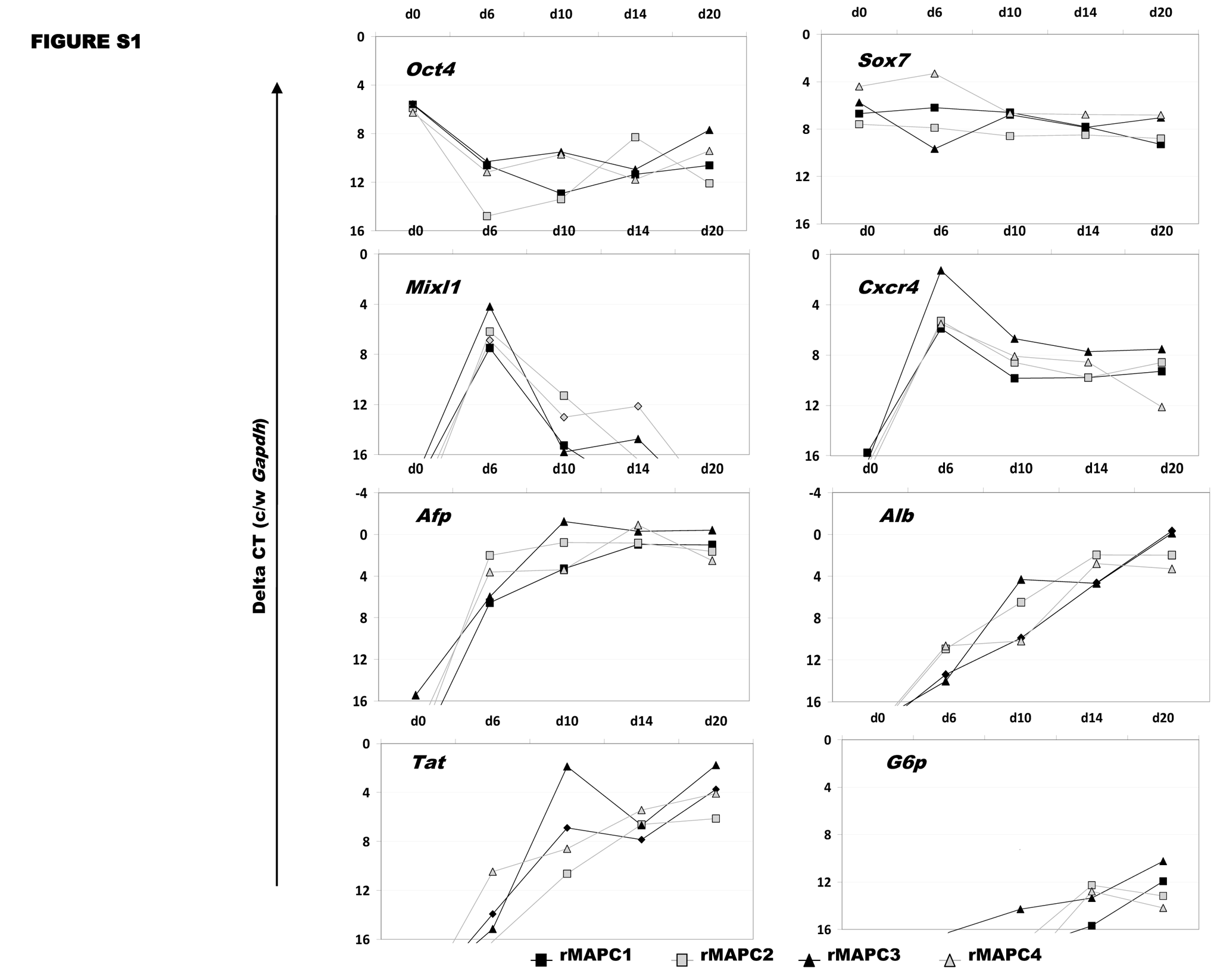

Supplement: Figure S1 — Quantitative RT-PCR method was used to evaluate expression levels of Oct4, the primitive/visceral endoderm specific gene, Sox7, and levels of some genes expressed during PS/ME/DE (Mixl1, Cxcr4), and hepatoblast/hepatocyte commitment (Afp, Alb, Tat and G6pc). Shown are mean DeltaCT values of >3 for rMAPC-1 (data also in Figure 2 and Table S1) and rMAPC-2 (data also in Table S1), and two additional cell lines (n = 1) different experiments on days 0, 6, 10, 14 and 20. (0.30 MB TIF) [file pone.0012101.s001.tif]
