# Supplementary material for: Human Embryonic and Rat Adult Stem Cells with Primitive Endoderm-Like Phenotype Can Be Fated to Definitive Endoderm, and Finally Hepatocyte-Like Cells
Source: PLoS One. 2010 Aug 11;5(8):e12101. doi: 10.1371/journal.pone.0012101 (PMC2920330; doi:10.1371/journal.pone.0012101)
Supplement: Table S1 — RT-qPCR analysis of gene expression in undifferentiated human ESC H9 and HSF6 (S1A and S1B) and rat MAPC-1 and rMAPC-2 (S1C and S1D), and during differentiation towards hepatocyte-like cells, using the protocol described in Figure 1, as well as values in fetal (third trimester) and adult human hepatocytes, and fetal (E15) and adult rat liver. Shown are mean DeltaCT values + s.d. (n>3). NE = not expressed (DeltaCT >16). − = not assessed. # = peak expression of MIXL1 on day 2, by day 6 expression already back to baseline. Some of these data are also shown in Figures 2 and S1. (0.23 MB DOC) [file pone.0012101.s002.doc]

**Table S1A Human ESC H9 Table S1B Human ESC HSF6**

|  | d0 | d6 | d10 | d14 | d20 |  | d0 | d6 | d10 | d14 | d20 |  | Fetal | Adult |
| --- | --- | --- | --- | --- | --- | --- | --- | --- | --- | --- | --- | --- | --- | --- |
| *OCT4* | 2.2 + 0.9 | 1.2 + 1.6 | 4.0 + 3.5 | 8.5 + 2.8 | 15.0 + 5.9 |  | 3.6 + 1.0 | 4.5 + 1.8 | 8.9 + 3.5 | 13.5 + 3.8 | 15.9 + 2.6 |  | 3.5 | 14.0 |
| *MIXL1* | 8.6 + 1.3 | 3.9 + 1.0 | 9.5 + 0.4 | 9.9 + 1.6 | 9.7 + 2.5 |  | 15.2 + 0.4 | NE # | NE | NE | NE |  | 1.2 | 12.8 |
| *EOMES* | 10.7 + 2.7 | 2.5 + 1.4 | 7.0 + 1.4 | 6.7 + 1.7 | 15.5 + 2.5 |  | 7.3 + 1.0 | 4.2 + 0.2 | 10.1 + 2.9 | 11.0 + 0.7 | 13.1 + 2.4 |  | 13.9 | 14.3 |
| *BRACHYURY* | 10.4 + 2.5 | 5.9 + 1.3 | 9.7 + 1.7 | 12.3 + 1.0 | 13.4 + 0.8 |  | 13.1 + 1.4 | 11.9 + 1.7 | 13.3 + 3.7 | NE | NE |  | 15.0 | NE |
| *SOX7* | 11.7 + 1.9 | 12.6 + 1.1 | 9.7 + 1.3 | 10.7 + 1.1 | 9.7 + 0.9 |  | - | - | - | - | - |  | 2.1 | 8.5 |
| *GSC* | 13.2 + 3.9 | 6.6 + 1.8 | 12.8 + 2.4 | 11.8 + 2.7 | 12.7 + 1.6 |  | 13.1 + 0.4 | 10.2 + 1.2 | 12.5 + 1.5 | NE | NE |  | 6.5 | NE |
| *SOX17* | 7.9 + 2.8 | 4.5 + 2.0 | 4.6 + 1.4 | 6.5 + 2.0 | 7.7 + 1.4 |  | 12.6 + 0.8 | 7.4 + 0.6 | 9.9 + 0.7 | 11.0 + 1.8 | 10.9 + 1.9 |  | 3.0 | 11.6 |
| *FOXA2* | 12.9 + 3.8 | 8.5 + 0.8 | 6.7 + 1.7 | 7.8 + 1.9 | 9.3 + 1.4 |  | 13.5 + 1.0 | 9.7 + 4.2 | 11.3 + 2.2 | 13.6 + 3.4 | 12.5 + 2.6 |  | NE | 7.6 |
| *CXCR4* | 6.3 + 2.5 | 3.9 + 1.1 | 6.4 + 1.4 | 4.7 + 1.5 | 6.5 + 1.0 |  | 13.9 + 0.5 | 9.7 + 2.2 | 13.6 + 1.0 | 14.1 + 0.9 | 13.9 + 0.4 |  | 1.3 | 9.2 |
| *E-CADHERIN* | 7.7 + 2.1 | 10.2 + 3.0 | 9.6 + 2.7 | 9.6 + 3.5 | 7.7 + 2.8 |  | 10.3 + 0.8 | 9.5 + 1.8 | 7.2 + 1 | 6.5 + 1.1 | 6.5 + 0.5 |  | NE | 2.0 |
| *PROX1* | 10.0 + 0.5 | 8.4 + 0.2 | 6.2 + 0.1 | 5.3 + 1.6 | 5.8 + 0.5 |  | 12.7 + 0.0 | 9.5 + 1.9 | 7.5 + 1.3 | 5.6 + 0.8 | 5.8 + 0.5 |  | 2.2 | -0.3 |
| *HNF1α* | 14.4 + 0.2 | 13.6 + 1.3 | 6.8 + 1.5 | 8.7 + 2.0 | 7.9 + 0.9 |  | NE | 14.5 + 2.0 | 10.1 + 1.9 | 9.2 + 1.5 | 9.3 + 0.6 |  | 1.7 | 4.1 |
| *HNF1β* | 8.4 + 0.7 | 6.2 + 1.2 | 4.4 + 0.1 | 5.5 + 1.2 | 5.2 + 2.4 |  | 12.1 + 1.3 | 8.8 + 1.6 | 6.4 + 2.1 | 6.9 + 1.4 | 7.6 + 1.6 |  | 11.4 | 3.2 |
| *HNF4α* | 7.3 + 2.5 | 7.2 + 1.1 | 4.3 + 1.4 | 5.6 + 1.5 | 5.6 + 2.8 |  | 12.3 + 2.2 | 11.2 + 1.2 | 10.0 + 2.8 | 9.1 + 2.3 | 8.7 + 1.3 |  | 13.9 | 1.7 |
| *HNF6* | 12.0 + 1.1 | 10.7 + 0.3 | 8.4 + 0.6 | 12.2 + 2.2 | 9.6 + 2.6 |  | 12.0 + 0.6 | 9.2 + 0.4 | 6.3 + 0.8 | 5.1 + 1.0 | 4.5 + 0.6 |  | 13.8 | 8.7 |
| *AFP* | 12.5 + 1.0 | 10.8 + 2.3 | -2.4 + 2.0 | -3.8 + 0.7 | -2.7 + 1.4 |  | NE | NE | 3.4 + 3.9 | 0.6 + 4.2 | -0.8 + 3.0 |  | 8.0 | 6.2 |
| *TTR* | 12.6 + 0.2 | 11.2 + 0.4 | -1.7 + 2.4 | -3.1 + 0.6 | - 1.8 + 1.6 |  | NE | 10.9 + 1.9 | 1.2 + 1.7 | -0.2 + 1.5 | -0.1 + 0.8 |  | 11.3 | -2.9 |
| *ALB* | NE | NE | 9.8 + 1.5 | 6.9 + 2.4 | -1.7 + 1.9 |  | NE | NE | 10.4 + 2.1 | 5.0 + 0.5 | 2.3 + 0.3 |  | 3.5 | -9.6 |
| *KRT7* | 8.1 + 2.1 | 8.0 + 3.9 | 2.7 + 0.8 | 4.3 + 0.7 | 4.5 + 1.0 |  | - | - | - | - | - |  | - | 9.2 |
| *KRT18* | 4.9 + 0.3 | 6.8 + 1.8 | 5.2 + 1.4 | 5.2 + 0.8 | 3.7 + 1.8 |  | 6.4 + 1.9 | 4.1 + 2.7 | 4.6 + 2.2 | 3.3 +/ 3.1 | 4.7 + 2.5 |  | 15.1 | 4.7 |
| *KRT19* | 7.2 + 2.3 | 7.7 + 1.2 | 6.4 + 4.5 | 6.0 + 1.6 | 6.9 + 5.7 |  | 4.5 + 0.2 | 0.5 + 2.5 | -0.7 + 2.6 | -1.0 + 1.6 | -1.5 + 1.5 |  | 5.1 | 7.8 |
| *SOX9* | 5.5 + 0.9 | 5.1 + 0.6 | 3.6 + 1.6 | 4.6 + 1.3 | 4.3 + 2.2 |  | - | - | - | - | - |  | 2.1 | 7.0 |
| *AAT* | NE | 15.7 + 4.1 | 8.0 + 3.6 | 5.7 + 1.5 | 1.4 + 0.9 |  | NE | NE | 3.9 + 1.6 | -1.5 + 1.2 | 1.3 + 1.5 |  | 8.4 | -3.8 |
| *G6PC* | NE | NE | 15.2 + 3.5 | NE | 15.8 + 0.5 |  | NE | 15.7 + 0.2 | 13.7 + 1.1 | 12.0 + 1.5 | 9.5 + 1.6 |  | 2.4 | 0.3 |
| *CX32* | NE | NE | 8.7 + 2.8 | 12.5 + 3.7 | 10.0 + 1.9 |  | NE | NE | 8.9 + 4.1 | 8.0 + 2.9 | 6.7 + 3.5 |  | 10.7 | 6.5 |
| *CYP3A4/5/7* | NE | 14.7 + 0.6 | 12.3 + 1.8 | 14.6 + 0.6 | 12.5 + 1.8 |  | NE | NE | 14.9 + 1.5 | 9.7 + 2.0 | 8.5 + 2.2 |  | 15.3 | -3.4 |
| *CYP3A7* | NE | 11.4 + 2.7 | 7.8 + 1.4 | 11.3 + 1.0 | 6.6 + 2.9 |  | NE | NE | 13.5 + 3.4 | 7.4 + 2.7 | 5.6 + 2.5 |  | 14.7 | 5.5 |
| *CYP7A1* | NE | NE | NE | 15.9 + 1.0 | 10.9 + 0.3 |  | NE | 12.7 + 3.0 | NE | 15.9 + 4.3 | 11.7 + 2.7 |  | NE | 7.8 |
| *UGT1A1* | - | - | - | - | - |  | NE | NE | NE | 12.3 + 2.0 | 10.7 + 2.2 |  | - | - |
| *GSTα* | 15.1 + 1.1 | 11.1 + 2.1 | 12.8 + 1.2 | 7.5 + 1.5 | 3.4 + 1.3 |  | 11.5 + 1.8 | 10.0 + 2.7 | 5.7 + 4.1 | 3.7 + 4.1 | 4.6 + 6.0 |  | - | 1.3 |
| *GSTµ* | 6.1 + 1.2 | 5.4 + 0.6 | 4.7 + 1.0 | 6.2 + 0.6 | 3.3 + 0.1 |  | 6.5 + 3.7 | 5.1 + 2.4 | 4.7 + 3.3 | 4.1 + 3.3 | 4.6 + 4.2 |  | - | 4.6 |
| *GSTπ* | 3.8 + 0.5 | 3.2 + 0.1 | 2.1 + 2.2 | 4.1 + 0.7 | 2.4 + 0.9 |  | 10.9 + 1.1 | 8.2 + 4.0 | 6.4 + 5.6 | 6.4 + 4.8 | 5.1 + 4.4 |  | - | 11.7 |
| *FACTOR V* | 14.8 + 0.7 | 13.8 + 4.3 | 12.4 + 0.3 | 10.2 + 1.0 | 9.4 + 0.8 |  | NE | - | - | 8.0 + 4.3 | 7.0 + 4.2 |  | 3.8 | -1.2 |
| *FACTOR VII* | 10.7 + 1.0 | 9.1 + 0.4 | 6.2 + 0.7 | 6.4 + 0.5 | 6.1 + 0.5 |  | NE | - | - | 8.1 + 2.2 | 8.4 + 1.9 |  | 4.9 | -0.8 |
| *PROTEIN C* | 12.5 + 0.4 | 13.6 + 1.1 | 11.9 + 0.9 | 11.2 + 2.8 | 8.8 + 2.8 |  | 14.7 + 2.0 | - | - | 6.2 + 1.5 | 5.8 + 1.4 |  | 3.4 | 2.0 |
| *GGCX* | 10.5 + 1.6 | 8.2 + 0.5 | 7.2 + 1.1 | 7.5 + 0.8 | 7.9 + 0.3 |  | NE | - | - | 8.7 + 2.1 | 8.0 + 1.8 |  | NE | 3.8 |
| *PTF1A* | NE | 15.1 + 2.1 | 14.8 + 3.1 | NE | NE |  | - | - | - | - | - |  | - | 14.6 |
| *NKX6.1* | 14.9 + 0.7 | 15.2 + 2.7 | 14.5 + 0.7 | NE | 14.5 + 1.3 |  | - | - | - | - | - |  | - | NE |
| *PDX1* | 11.8 + 1.2 | 10.8 + 3.2 | 11.0 + 4.3 | 11.4 + 0.7 | 10.8 + 3.2 |  | NE | NE | NE | 13.8 + 2.8 | 14.8 + 3.3 |  | - | - |
| *NGN3* | NE | NE | NE | NE | NE |  | - | - | - | - | - |  | - | - |

**Table S1C Rat MAPC-1 Table S1D Rat MAPC-2**

|  | d0 | d6 | d10 | d14 (*d16) | d20 |  | d0 | d6 | d10 | d14 | d20 |  | Fetal | Adult |
| --- | --- | --- | --- | --- | --- | --- | --- | --- | --- | --- | --- | --- | --- | --- |
| *Oct4* | 5.6 + 0.9 | 10.6 + 2.0 | 12.9 + 0.8 | 11.4 + 0.9* | 10.6 + 1.2 |  | 5.9 + 1.0 | 14.8 + 1.5 | 13.4 + 2.5 | 8.3 + 1.1 | 12.1 + 3.9 |  | NE | 13.7 |
| *Sox7* | 6.7 + 2.1 | 6.2 + 1.5 | 6.6 + 0.3 | 7.8 + 2.5* | 9.3 + 3.0 |  | 7.6 + 0.9 | 7.9 + 0.9 | 8.6 + 1.3 | 8.5 + 1.3 | 8.8 + 0.9 |  | 13.4 | - |
| *Sox17* | 2.5 + 1.3 | 3.1 + 1.9 | 5.8 + 0.9 | 8.5 + 0.2* | 7.9 + 2.0 |  | 3.8 + 1.9 | 6.6 + 2.0 | 6.3 + 1.6 | 5.8 + 1.5 | 6.1 + 1.5 |  | 10.4 | 11.0 |
| *Foxa2* | 4.5 + 0.5 | 3.0 + 0.8 | 3.5 + 0.2 | 4.5 + 0.9* | 4.4 + 0.5 |  | 6.9 + 0.1 | 5.3 + 0.5 | 5.7 + 0.2 | 5.2 + 0.6 | 7.2 + 0.7 |  | 8.6 | 4.9 |
| *Mixl1* | NE | 7.5 + 2.0 | 15.3 + 0.5 | NE* | NE |  | NE | 6.2 + 1.6 | 11.3 + 1.1 | NE | NE |  | NE | - |
| *Eomes* | 8.7 + 1.0 | 3.7 + 1.4 | 9.2 + 0.7 | 9.9 + 0.1* | 9.4 + 0.3 |  | 8.5 + 0.2 | 4.5 + 0.2 | 8.0 + 0.5 | 9.9 + 1.0 | 10.1 + 0.2 |  | 15.8 | - |
| *Gsc* | 12.9 + 1.4 | 6.3 + 1.9 | 14.9 + 1.2 | 13.3 + 1.1* | 15.8 + 1.1 |  | 14.5 + 1.0 | 6.2 + 0.2 | NE | NE | NE |  | NE | - |
| *Cxcr4* | 15.7 + 1.8 | 5.9 + 1.5 | 9.8 + 0.9 | 9.8 + 1.0* | 9.3 + 0.6 |  | NE | 5.3 + 1.3 | 8.6 + 0.8 | 9.8 + 0.7 | 8.6 + 1.6 |  | 9.4 | - |
| *Tm4sf2* | NE | 2.6 + 0.9 | 6.7 + 0.4 | 8.0 + 1.2* | 7.2 + 0.9 |  | NE | 3.2 + 0.4 | 6.4 + 1.1 | 7.5 + 0.4 | 9.1 + 0.4 |  | 4.7 | - |
| *Tmprss2* | 12.8 + 0.4 | 2.6 + 0.4 | 1.9 + 0.4 | 2.7 + 0.5 | 2.8 + 0.2 |  | - | - | - | - | - |  | 14.4 | - |
| *Thbd* | 9.6 + 0.8 | 8.3 + 1.4 | 8.4 + 1.3 | 7.9 + 1.0 | 8.0 + 1.0 |  | - | - | - | - | - |  | - | - |
| *E-cadherin* | 6.2 + 0.3 | 1.1 + 0.9 | 1.2 + 0.5 | 2.1 + 1.0 | 2.2 + 0.9 |  | 6.1 + 0.5 | 1.9 + 0.2 | 1.9 + 0.1 | 2.2 + 0.1 | 2.6 + 0.1 |  | - | - |
| *Prox1* | 9.1 + 0.4 | 5.3 + 0.8 | 4.2 + 1.3 | 5.5 + 1.6* | 4.8 + 0.9 |  | 8.7 + 0.4 | 5.2 + 0.7 | 4.8 + 0.2 | 5.0 + 0.5 | 5.4 + 0.4 |  | 6.3 | 3.7 |
| *Hnf1α* | 11.5 + 0.5 | 8.3 + 0.7 | 7.7 + 0.7 | 8.1 + 0.4* | 7.7 + 0.5 |  | 11.6 + 1.1 | 8.2 + 0.9 | 8.3 + 1.9 | 7.8 + 0.8 | 7.7 + 1.2 |  | 8.9 | 7.6 |
| *Hnf1β* | 6.9 + 1.1 | 6.7 + 0.9 | 6.4 + 1.2 | 7.1 + 0.8* | 7.0 + 0.8 |  | 8.4 + 1.2 | 6.1 + 0.4 | 6.7 + 0.2 | 6.9 + 0.8 | 6.5 + 0.2 |  | 11.5 | 7.4 |
| *Hnf4α* | 15.2 + 1.5 | 6.2 + 0.8 | 7.7 + 1.7 | 8.0 + 1.3* | 6.9 + 1.5 |  | 13.3 + 0.8 | 7.0 + 0.5 | 6.1 + 0.6 | 7.7 + 1.4 | 6.9 + 1.9 |  | 4.8 | 2.5 |
| *Hnf6* | NE | 9.2 + 1.4 | 5.3 + 1.2 | 4.9 + 0.2* | 5.2 + 0.8 |  | NE | 7.8 + 1.1 | 5.0 + 1.3 | 5.5 + 0.5 | 4.6 + 1.0 |  | 9.0 | 4.4 |
| *Afp* | NE | 5.2 + 2.0 | 0.0 + 0.7 | -0.4 + 1.0* | -0.2 + 0.8 |  | NE | 2.0 + 0.7 | 0.8 + 0.7 | 0.8 + 0.9 | 1.6 + 0.8 |  | -2.4 | 8.8 |
| *Ttr* | 15.5 + 0.9 | 2.9 + 1.3 | 0.3 + 0.6 | 0.4 + 0.9* | 0.3 + 0.6 |  | NE | 2.8 + 1.2 | 0.6 + 1.9 | 1.8 + 1.2 | 1.5 + 1.8 |  | -0.3 | -2.5 |
| *Krt19* | 7.4 + 0.2 | 3.8 + 0.5 | 3.1 + 0.5 | 2.7 + 0.5* | 1.8 + 0.2 |  | 8.8 + 0.6 | 5.0 + 0.2 | 4.7 + 0.6 | 3.1 + 0.7 | 3.4 + 0.3 |  | 9.2 | 11.2 |
| *Krt7* | NE | 6.8 + 1.3 | 8.7 + 1.3 | 5.9 + 0.3 | 4.6 + 0.4 |  | - | - | - | - | - |  | NE | - |
| *Sox9* | NE | NE | NE | NE | NE |  | - | - | - | - | - |  | - | - |
| *Alb* | NE | 13.9 + 1.2 | 8.9 + 1.7 | 6.2 + 1.5* | 1.1 + 1.8 |  | NE | 11.0 + 0.6 | 6.5 + 0.2 | 2.0 + 0.4 | 2.0 + 0.8 |  | -2.9 | -5.7 |
| *Krt18* | 0.8 + 2.2 | -0.6 + 0.8 | 0.5 + 0.3 | 0.8 + 0.4* | 0.7 + 0.2 |  | 3.9 + 1.1 | -1.3 + 0.2 | 0.1 + 1.1 | 1.0 + 0.5 | 0.4 + 1.4 |  | 5.0 | 3.7 |
| *Aat* | NE | 9.9 + 3.6 | 2.6 + 0.5 | 0.8 + 1.1* | 1.3 + 1.0 |  | NE | 9.4 + 2.1 | 3.1 + 0.6 | 2.4 + 0.4 | 1.9 + 0.6 |  | -0.6 | -2.8 |
| *Tat* | NE | 13.0 + 2.3 | 9.3 + 1.7 | 7.4 + 0.8* | 4.3 + 1.1 |  | NE | NE | 10.6 + 0.3 | 6.6 + 0.4 | 6.1 + 0.4 |  | 15.6 | 0.8 |
| *G6pc* | NE | NE | NE | 14.6 + 1.3* | 11.4 + 1.1 |  | NE | NE | NE | 12.3 + 1.6 | 13.2 + 0.9 |  | 14.0 | 2.6 |
| *Pepck1* | NE | 9.0 + 0.6 | NE | 12.9 + 0.9 | 11.7 + 1.2 |  | NE | 11.7 + 2.2 | NE | 14.8 + 4.1 | 11.0 + 1.9 |  | 12.5 | 8.1 |
| *Mrp2* | NE | 15.0 + 1.4 | 12.6 + 2.0 | 11.6 + 2.1* | 10.7 + 1.5 |  | NE | 14.5 + 0.4 | 10.0 + 0.2 | 11.2 + 0.7 | 10.4 + 0.3 |  | 8.0 | 1.7 |
| *Bsep* | NE | NE | 15.6 + 1.1 | 15.4 + 0.5* | 13.2 + 1.4 |  | - | - | - | - | - |  | 12.1 | 3.0 |
| *Arg1* | 14.7 + 0.6 | 9.2 + 0.7 | 7.8 + 0.7 | 8.0 + 1.3* | 8.1 + 0.9 |  | - | - | - | - | - |  | 6.6 | 2.1 |
| *Cx32* | NE | NE | NE | 13,9 + 0,7* | 12,8+0,9 |  | NE | NE | 15.7 + 2.2 | 14.0 + 1.3 | 13.3 + 0.9 |  | 14.0 | 9.3 |
| *Cyp1a2* | NE | NE | NE | NE | 16.0 + 1.6 |  | - | - | - | - | - |  | NE | 15.5 |
| *Gstα* | 8.6 + 0.9 | 10.6 + 1.7 | 10.7 + 1.2 | 10.3 + 1.5 | 9.8 + 0.7 |  | 9.0 + 0.3 | 10.2 + 0.2 | 9.8 + 0.5 | 8.9 + 0.4 | 9.6 + 0.1 |  | 5.7 | - |
| *Gstµ* | 6.4 + 1.8 | 5.3 + 0.9 | 5.3 + 1.0 | 4.7 + 0.7 | 4.1 + 0.5 |  | 7.3 + 0.6 | 5.9 + 0.1 | 3.2 + 0.1 | 4.0 + 0.2 | 4.3 + 0.2 |  | 3.6 | 0.3 |
| *Gstπ* | 4.7 + 1.0 | 1.9 + 1.0 | 1.6 + 0.9 | 1.6 + 0.7 | 1.2 + 0.7 |  | 6.3 + 1.2 | 1.4 + 1.0 | 0.0 + 0.3 | 1.1 + 0.2 | 1.8 + 0.1 |  | 2.8 | 8.6 |
| *Factor V* | NE | 10.5 + 3.5 | 9.9 + 3.1 | 8.8 + 2.8 | 8.4 + 2.5 |  | - | - | - | - | - |  | 7.5 | 6.3 |
| *Factor VII* | 13.1 + 0.1 | 13.7 + 1.6 | 13.8 + 2.6 | 14.6 + 2.2 | 13.1 + 1.8 |  | - | - | - | - | - |  | 8.8 | 13.9 |
| *Protein C* | 15.9 + 0.9 | 5.7 + 1.1 | 5.5 + 1.5 | 5.0 + 0.9 | 6.7 + 0.8 |  | - | - | - | - | - |  | 5.8 | 11.8 |
| *Ggcx* | 13.2 + 3.2 | 10.3 + 2.3 | 9.3 + 1.7 | 9.6 + 1.6 | 9.3 + 2.2 |  | - | - | - | - | - |  | 9.2 | 9.9 |
| *Ptf1a* | NE | NE | NE | NE | NE |  | - | - | - | - | - |  | - | - |
| *Nkx6.1* | NE | 14.9 + 0.3 | NE | NE | NE |  | - | - | - | - | - |  | - | - |
| *Pdx1* | NE | 14.4 + 2.7 | 14.3 + 1.3 | 14.6 + 1.0 | 14.8 + 0.7 |  | - | - | - | - | - |  | 15.7 | - |
| *Ngn3* | NE | NE | NE | NE | NE |  | - | - | - | - | - |  | - | - |
