# Supplementary material for: Human Embryonic and Rat Adult Stem Cells with Primitive Endoderm-Like Phenotype Can Be Fated to Definitive Endoderm, and Finally Hepatocyte-Like Cells
Source: PLoS One. 2010 Aug 11;5(8):e12101. doi: 10.1371/journal.pone.0012101 (PMC2920330; doi:10.1371/journal.pone.0012101)
Supplement: Table S2 — Albumin production by hESC-H9, hESC-HSF6, rMAPC-1 and rMAPC-2 was measured in culture supernatants of undifferentiated stem cells as well as at different time points of differentiation towards hepatocyte-like cells and in supernatants of primary hepatocytes. Data are shown as mean concentrations after 48 h (ng/ml/48 h) + s.d. (n>3). − = not assessed. The mean values op hESC-H9 and rMAPC-1 are also shown in Figure 4A. (0.05 MB DOC) [file pone.0012101.s003.doc]

**Table S2: Albumin secretion**

|  | d0 | d6 | d10 | d14 | d20 | Primary hepatocytes |
| --- | --- | --- | --- | --- | --- | --- |
| hESC-H9 | 0 | 1.1 + 1.9 | 0.1 + 1.8 | 1.5 + 3.8 | 1,270.4 + 833.8 | 48,252 + 12,465 |
| hESC-HSF6 | - | - | - | 36.5 + 14.1 | 2,750 + 634 |
| rMAPC-1 | 0 | 0 | 0 | 4.3 + 4.0 | 617 + 815 | 105,602 + 73,631 |
| rMAPC-2 | 0 | 0.0 + 0.0 | 2.5 + 3.6 | 842.3 + 495.0 | 3,441.1 + 1343.2 |
