# Supplementary material for: Human Embryonic and Rat Adult Stem Cells with Primitive Endoderm-Like Phenotype Can Be Fated to Definitive Endoderm, and Finally Hepatocyte-Like Cells
Source: PLoS One. 2010 Aug 11;5(8):e12101. doi: 10.1371/journal.pone.0012101 (PMC2920330; doi:10.1371/journal.pone.0012101)
Supplement: Table S3 — RT-qPCR analysis of expression of PS/ME/DE genes on d0 and d6 in rMAPC-1 treated with no, 1 ng/ml or 100 ng/ml Activin-A. Data are all from paired experiments, and are shown as DeltaCT compared with Gapdh. Shown are mean DeltaCT values + s.d. (n >3, except * n = 2). # = p<0.05 versus 100 ng/ml Activin-A. (0.06 MB DOC) [file pone.0012101.s004.doc]

**Table S3: Expression of PS/ME/DE genes in rMAPC d6-progeny exposed to different concentrations of Activin-A**

|  | d0 |  | No Activin-A  No Wnt3a  d6 |  | Activin-A 1 ng/mL Wnt3a 50 ng/ml  d6 |  | Activin-A 100 ng/ml  Wnt3a 50 ng/ml  d6 |
| --- | --- | --- | --- | --- | --- | --- | --- |
|  |  |  |
| *Oct4* | 5.6 + 0.9 |  | 10.3 + 1.0 |  | 8.1 + 1.1 |  | 11.3 + 2.6 |
| *Mixl1* | NE |  | NE# |  | NE#* |  | 8.2 + 1.9 |
| *Eomes* | 8.7 + 1.0 |  | 5.4 + 1.4 |  | 3.1 + 0.0* |  | 3.8 + 2.2 |
| *Cxcr4* | 15.8 + 1.8 |  | 8.7 + 2.3# |  | 7.9 + 2.0# |  | 6.2 + 2.0 |
| *Gsc* | 12.9 + 1.4 |  | 10.0 + 1.6# |  | 8.3 + 1.5# |  | 6.1 + 2.6 |
| *Tm4sf2* | NE |  | 11.5 + 1.2# |  | 10.5 + 0.2# |  | 3.4 + 0.4 |
