# Supplementary material for: Human Embryonic and Rat Adult Stem Cells with Primitive Endoderm-Like Phenotype Can Be Fated to Definitive Endoderm, and Finally Hepatocyte-Like Cells
Source: PLoS One. 2010 Aug 11;5(8):e12101. doi: 10.1371/journal.pone.0012101 (PMC2920330; doi:10.1371/journal.pone.0012101)
Supplement: Table S4 — Primer list. (0.12 MB DOC) [file pone.0012101.s005.doc]

**Table S4A:**  HUMAN PRIMERS

| **Genes** | **Forward sequence** | **Reverse sequence** |
| --- | --- | --- |
| **AAT (H9)** | TTTAAAGGCAAATGGGAGAG | CCTAAACGCTTCATCATAGG |
| **AAT (HSF6)** | GTCAAGGACACCGAGGAAGA | TATTTCATCAGCAGCACCCA |
| **AFP (H9)** | CCTACAATTCTTCTTTGGGCT | AGTAACAGTTATGGCTTGGA |
| **AFP (HSF6)** | AAATGCGTTTCTCGTTGCTT | GCCACAGGCCAATAGTTTGT |
| **ALB** | TGGCACAATGAAGTGGGTAA | CTGAGCAAAGGCAATCAACA |
| **BRACHYURY** | AATTGGTCCAGCCTTGGAAT | ACAGGCTGGGGTACTGACTG |
| **CX32** | CCTTGCTCAGTGGCGTGAA | GCTGGAGTGTGTTGCAGATG |
| **CXCR4** | AACTTCAGTTTGTTGGCTGC | GAAACAGGGTTCCTTCATGG |
| **CYP3A4/5/7** | AAGTCGCCTCGAAGATACACA | AAGGAGAGAACACTGCTCGTG |
| **CYP3A7** | TGCTTTGTCCTTCCGTAAGGG | CAGCATAGGCTGTTGACAGTC |
| **CYP7A1** | CTGAGGCTTTCCAGTGCCT | AGGTAGTCTTTGTCTTCCCGT |
| **E-CADHERIN (HSF6)** | CGAGAGCTACACGTTCACGG | GTGTCGAGGGAAAAATAGGCTG |
| **E-CADHERIN (H9)** | CGAACTATATTCTTCTGTGAGAGG | GATAGATTCTTGGGTTGGGTC |
| **EOMES** | AACAACACCCAGATGATAGTC | TCATAGTTGTCTCTGAAGCCT |
| **FACTOR V (H9)** | CAAATCGTGCTACTATTTGCTG | CTGTTATATCTGGCATTGTCCC |
| **FACTOR V (HSF6)** | GACAGAAGCGGCACAGCTAA | GTCCTGAAATGGTAGATTGTGGT |
| **FACTOR VII** | CAAACCCCAAGGCCGAATTG | CGCGATCAGGTTCCTCCAG |
| **FOXA2** | ATTGCTGGTCGTTTGTTGTG | TACGTGTTCATGCCGTTCAT |
| **G6PC** | GTGTCCGTGATCGCAGACC | GACGAGGTTGAGCCAGTCTC |
| **GAPDH (H9)** | TGGTATCGTGGAAGGACTCATGAC | ATGCCAGTGAGCTTCCCGTTCAGC |
| **GAPDH (HSF6)** | GAGTCAACGGATTTGGTCGT | GACAAGCTTCCCGTTCTCAG |
| **GGCX** | GTGTTTCTCCTGGACAAGAC | AGACAACAGCAGTTTGAAGG |
| **GSC** | TCTCAACCAGCTGCACTGTC | CCAGACCTCCACTTTCTCCTC |
| **GSTα** | TCTGCCCGTATGTCCACCT | GCTCCTCGACGTAGTAGAGAAGT |
| **GSTμ** | CAGAATTTGAGAAACTGAAGCC | ACAGCCATCTTTGAGAACAC |
| **GSTπ** | CCCTACACCGTGGTCTATTTCC | GAGGCTTTGAGTGAGCCCT |
| **HNF1α** | GTGGCGAAGATGGTCAAGTCC | CCCTTGTTGAGGTGTTGGG |
| **HNF1β** | AGGCCACAATCTCCTCTCAC | TTGCTGGGGATTATGGTGGGA |
| **HNF4α** | TGTACTCCTGCAGATTTAGCC | CTGTCCTCATAGCTTGACCT |
| **HNF6** | CAAACCCTGGAGCAAACTCAA | TGTGTTGCCTCTATCCTTCCC |
| **KRT7** | CTCAATGAGACGGAGTTGAC | CCGGTTCATCTCTGAAATCTC |
| **KRT18** | TGATGACACCAATATCACACGAC | TACCTCCACGGTCAACCCA |
| **KRT19** | GAACCATGAGGAGGAAATCAG | CATGTCACTCAGGATCTTGG |
| **MIXL1** | GGATCCAGGTATGGTTCCAG | CATGAGTCCAGCTTTGAACC |
| **NGN3** | TCTCTATTCTTTTGCGCCGG | CTTGGACAGTGGGCGCAC |
| **NKX6.1** | TCAGGTCAAGGTCTGGTTCC | TCAACAGCTGCGTGATTTTC |
| **OCT4 (H9)** | GATGGCGTACTGTGGGCCC | TGGGACTCCTCCGGGTTTTG |
| **OCT4 (HSF6)** | CTTCGCAAGCCCTCATTTC | CCTTGGAAGCTTAGCCAGGT |
| **PDX1 (H9)** | TCCACCTTGGGACCTGTTTA | GTGTGTTAGGGAGCCTTCCA |
| **PDX1 (HSF6)** | CATTGGAAGGCTCCCTAACACA | GGCATCAATTTCACGGGATC |
| **PROTEIN C** | CGAACTTGCAGTATCTCCAC | AGAACACTGAGTCAAGAGGA |
| **PROX1** | GGATGTTGAGTATTCAGTGGTGC | CTGGGAAATTATGGTTGCTCCT |
| **PTF1A** | ACGACTTCTTCACCGACCAG | TGGTGGCTAAGGAACTCCAC |
| **SOX7** | GCCTGTGCAACAAGAGTGAA | GTACCCTGGGTCTTTGGTCA |
| **SOX9** | CAAGACTCTGGGCAAGCTCTG | TCCGCTTGTCCGTTCTTCAC |
| **SOX17** | CGCACGGAATTTGAACAGTA | GGATCAGGGACCTGTCACAC |
| **TTR (H9)** | AAACCAGTGAGTCTGGAGAG | CTGTGAATACCACCTCTGCA |
| **TTR (HSF6)** | ATCCAAGTGTCCTCTGATGGT | GCCAAGTGCCTTCCAGTAAGA |
| **UGT1A1** | CAACTGCCTTCACCAAAATCCA | GCAAGATTCGATGGTCGGGTT |

**Table S4B:** RAT PRIMERS

| **Genes** | **Forward sequence** | **Reverse sequence** |
| --- | --- | --- |
| **Aat** | CAAACAAGGTCAGCCATTCTC | CAGCATCATTGTTGAAGACCC |
| **Afp** | ACCTGACAGGGAAGATGGTG | GCAGTGGTTGATACCGGAGT |
| **Alb** | TCTGCACACTCCCAGACAAG | AGTCACCCATCACCGTCTTC |
| **Arg1** | TATCGGAGCGCCTTTCTCTA | ACAGACCGTGGGTTCTTCAC |
| **Bsep** | CACTGGGTACATGTGGTGTCTCAT | ATGGCCAATATTCATAGCTGCTAAT |
| **Cxcr4** | GGATGGTGGTGTTCCAGTTC | TCCCCACGTAATACGGTAGC |
| **Cx32** | GACCACTCCCCCTACACAGA | CTTTCTTCCCCAGGCCTAAC |
| **Cyp1a2** | GTCCAGGAACACTATCAAGAC | ACTGTTTCAAATCCAGCTCC |
| **E-cadherin** | TGACAAGTACCTTCTCTACTCTC | AACAGATCCCTCAAAGACCT |
| **Eomes** | CCAGACCTTCACCTTCTCAG | GTGTACATGGAATCGTAGTTGTC |
| **Factor V** | CAATGCCAGATGTAACAGTC | TGTCAATATAAGCCTGCATCC |
| **Factor VII** | GCTTCTGCCCCCTAGACTTT | CCGCATGGGTACTCAACTTT |
| **Foxa2** | GCAGAACTCCATCCGTCATT | TCGAACATGTTGCCAGAGTC |
| **G6pc** | GATTCCGGTGCTTGAATGTC | AGGTGATGAGACAGTACCTC |
| **Gapdh** | Taqman ® Rodent Gapdh control reagents (Applied Biosystems 4308313) | |
| **Ggcx** | CCTTTCAGTTGACATTCATGGA | CAACACTAGCTTGAAGGGAC |
| **Gsc** | CCCGGTTCTGTACTGGTGTC | CCCACGTCTGGGTACTTTGT |
| **Gstα** | CAGGAGTGGAGTTTGATGAG | CCATAGAGGTCATATTTGGTGG |
| **Gstμ** | TACTTAATTGATGGATCGCGC | CTTCTGCTTCTCAAAGTCGG |
| **Gstπ** | GTGGATATGGTGAATGATGGG | TGCAAAGGAAATCTGGTTACC |
| **Hnf1α** | CAGCCACAACCATTCACATC | GCCATCTGGGTGGAGATAAA |
| **Hnf1β** | AGAACTCCCACATGTATGCA | CAGTGGACACTGTTTACTGG |
| **Hnf4α** | AAATGTGCAGGTGTTGACCA | CACGCTCCTCCTGAAGAATC |
| **Hnf6** | CTGTGAAACTCCCCCAGGTA | TCATCCCGCATAAGTGTGAA |
| **Krt7** | TTGACACCTTGAAGAACCAG | TCACAACAGAGATATTCACAGG |
| **Krt18** | TTTGCGAATTCTGTGGACAA | ACCTCGTGATGTTGGTGTCA |
| **Krt19** | CCACACTACGCAGATCCAGA | ATGCTGAGCTGAGACTGCAA |
| **Mixl1** | GGGAAGATTTCCTCCATCGT | CTGAGAACCAGATGTACAGAC |
| **Mrp2** | TAGTCTTCGCCGTACACTGAGC | ACATTCACATTTTTAATCTTCAAGGAGTT |
| **Ngn3** | GAGTGGGTGGGCGTACTCTA | TTGGAACTGAGCACTTCGTG |
| **Nkx6.1** | ACTTGGCAGGACCAGAGAGA | GGAACCAGACCTTGACCTGA |
| **Oct4** | CTGTAACCGGCGCCAGAA | TGCATGGGAGAGCCCAGA |
| **Pdx1** | TCTGCCTCTGGGACTCTTTC | GGGACCGCTCAAGTTTGTAA |
| **Pepck1** | ATATGACAACTGTTGGCTGG | AATGCTTTCTCGAAGTCCTC |
| **Ptf1a** | GTAACCAGGCCCAGAAGGTC | GAGGAGGGAGACCGTAGTCC |
| **Protein C** | AACTTCAAACGGGACATAGAC | AGATCATACTCACCAAGCCT |
| **Prox1** | GGGACAGCATCTGCATTTTT | AAAGGCATCATGGCATCTTC |
| **Sox7** | CAAGGATGAGAGGAAACGTC | CTCTGCCTCATCCACATAGG |
| **Sox9** | TAACGCCATCTTCAAGGC | CGATGTTGGAGATGACGTC |
| **Sox17** | GCAAGATGCTAGGCAAATCC | GTACTTGTAGTTGGGATGGTC |
| **Tat** | GGAAGCTAAGGATGTCATTCTG | GACCTCAATTCCCATAGACTC |
| **Thbd** | GCTATGATGGCTATGAGTTGG | GTAGTGCGTGGAGTTTACTG |
| **Tmprss2** | AGGTTTACTCATCTCAGAGG | AAAGCTGTTCTTGTATCCCA |
| **Tm4sf2** | CTGAAACTGTATGCCATGTTCC | ATCTTTGCCGTTGTAGTTCTG |
| **Ttr** | CAGCAGTGGTGCTGTAGGAGTA | GGGTAGAACTGGACACCAAATC |
