# Supplementary material for: Human Embryonic and Rat Adult Stem Cells with Primitive Endoderm-Like Phenotype Can Be Fated to Definitive Endoderm, and Finally Hepatocyte-Like Cells
Source: PLoS One. 2010 Aug 11;5(8):e12101. doi: 10.1371/journal.pone.0012101 (PMC2920330; doi:10.1371/journal.pone.0012101)
Supplement: Table S5 — Immunohistochemistry antibodies. (0.06 MB DOC) [file pone.0012101.s006.doc]

**Table S5**

Primary antibodies

| **Name** | **Company** | **Catalog no.** | **Dilution** | **Isotype** |
| --- | --- | --- | --- | --- |
| AFP | R &D Systems | MAB 1368 | 1:4000 | Mouse IgG_1_ |
| ALB | Dako | A0001 | 1:8000 | Rabbit IgG |
| FOXA2 | Santa Cruz. Biotech | SC6554 | 1:200 | Goat IgG |
| HNF4A | Abcam | AB41898 | 1:200 | Mouse IgG_2A_ |
| KRT18 (N16) | Santa Cruz. Biotech | SC31700 | 1:250 | Goat IgG |
| MIXL1 | Santa Cruz. Biotech | SC98665 | 1:100 | Rabbit IgG |
| OCT3/4 (N19) | Santa Cruz. Biotech | SC8628 | 1:1000 | Goat IgG |
| PEPCK (H800) | Santa Cruz. Biotech | SC32879 | 1:2000 | Rabbit IgG |
| SOX7 | R &D Systems | AF2766 | 1:20 | Goat IgG |
| SOX17 | R &D Systems | MAB 1924 | 1:300 | Mouse IgG_3_ |

**Isotypes (protein concentration identical to primary antibody)**

| Mouse IgG_1_ | BD Biosciences | 550878 |
| --- | --- | --- |
| Mouse IgG_2A_ | Sigma | M9144 |
| Goat IgG | Jackson Labs | JACK005-000-0020 |
| Rabbit serum | Dako | X0902 |

Secondary antibodies

| **Name** | **Company** | **Catalog no.** | **Dilution** |
| --- | --- | --- | --- |
| Chicken anti-rabbit Alexa 488 (green) | Invitrogen | A21441 | 1:500 |
| Goat anti-mouse Alexa 488 (green) | Invitrogen | A11017 | 1:500 |
| Rabbit anti-mouse Alexa 488 (green) | Invitrogen | A11059 | 1:500 |
| Donkey anti-goat Alexa 555 (red) | Invitrogen | A21432 | 1:500 |
| Goat anti-rabbit Alexa 555 (red) | Invitrogen | A21429 | 1:500 |
| Donkey anti-goat Alexa 633 (far red) | Invitrogen | A21082 | 1:500 |
| Goat anti-rabbit Alexa 633 (far red) | Invitrogen | A21071 | 1:500 |
| Hoechst (nuclear staining, blue) | Sigma | 33258 | 1:2000 |
